# Supplementary material for: Peroxisome Proliferator-Activated Receptor γ2 Controls the Rate of Adipose Tissue Lipid Storage and Determines Metabolic Flexibility
Source: Cell Rep. 2018 Aug 24;24(8):2005–2012.e7. doi: 10.1016/j.celrep.2018.07.063 (PMC6113930; doi:10.1016/j.celrep.2018.07.063)
Supplement: Document S1. Figures S1–S4 and Table S1 [file mmc1.pdf]

Cell Reports, Volume *24*

Supplemental Information

Peroxisome Proliferator-Activated Receptor γ2 Controls the Rate of Adipose Tissue Lipid Storage and Determines Metabolic Flexibility

Sam Virtue, Kasparas Petkevicius, José Maria Moreno-Navarrete, Benjamin Jenkins, Daniel Hart, Martin Dale, Albert Koulman, José Manuel Fernández-Real, and Antonio Vidal-Puig

Supplementary Results

Supplemental Figures

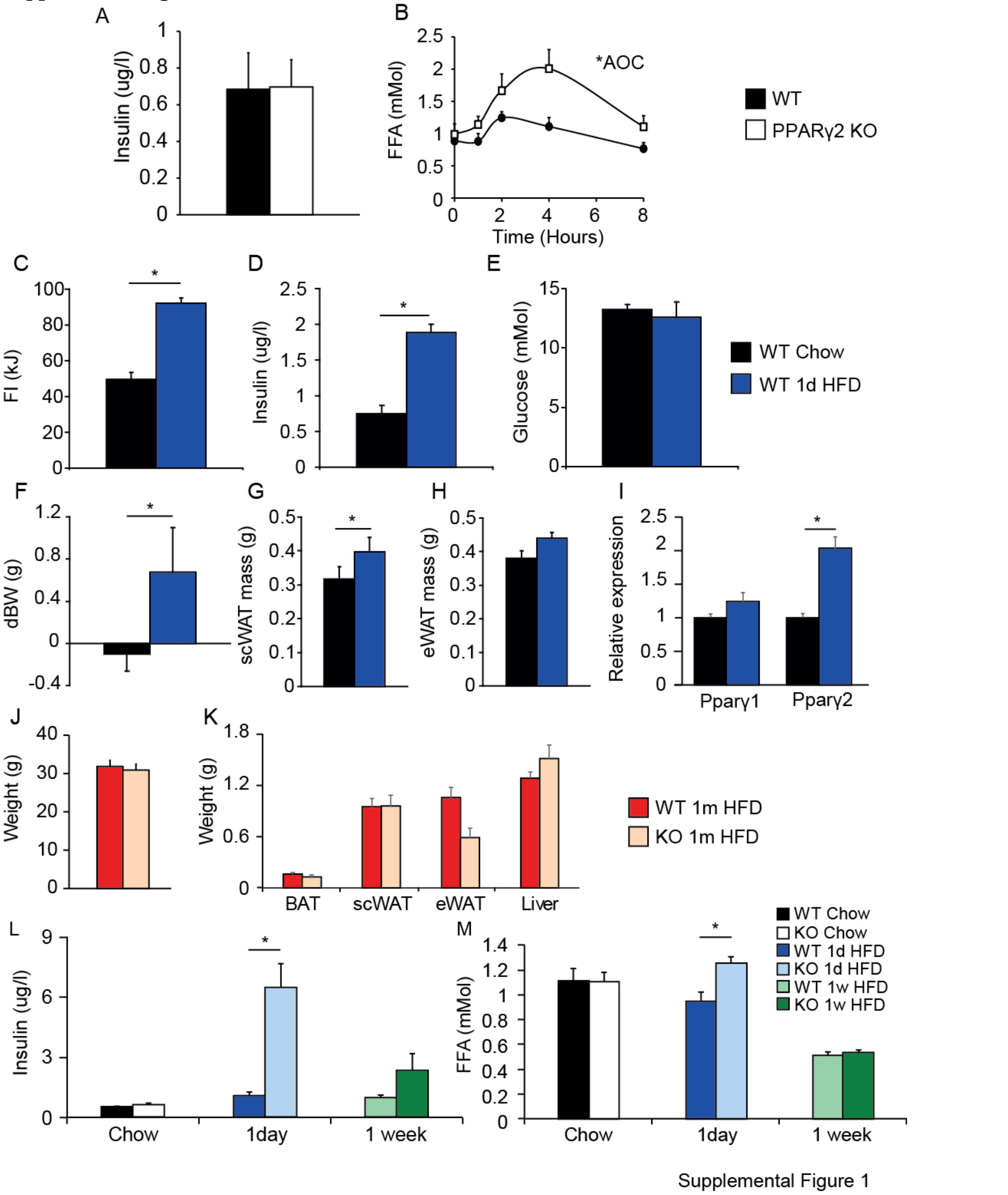

Figure S1 related to figures 1 and 2. A) Fasted insulin levels from chow fed PPARγ2 KO and WT mice N= 6 WT and 9 KO. B) FFA levels during a lipid tolerance test for WT and PPARγ2 KO mice. N=10 WT and 9 KO, 4 months old. *P<0.05 two-tailed students T-test. Metabolic and anthropomorphic characteristics of mice fed a high fat diet for one day (1dHFD) or chow controls; C) food intake (n=8 per group) D) serum insulin E) blood glucose (N=5 chow and 6 HFD) F) body weight change (dBW) G) subcutaneous white adipose tissue (WAT) mass H) epididymal WAT mass N=12 mice per group, C57bl/6 males, 10 weeks old. I) Gene expression in scWAT of 1dHFD fed mice or chow fed controls, N=6 mice per group, C57bl/6 males, 10 weeks old. J) Body weight and K) tissue weights for mice lacking PPARγ2 and wild-type controls following 1 month of high-fat feeding N=8 WT and 7 KO, 4 months of age. L) Insulin and M) FFA levels of a cohort of mice fed a chow control diet then switched to a high fat diet and blood samples measured after 0, 1 or 7 days of high-fat feeding. N= 7 mice per group, 3 months of age. All data represented as mean ±SEM.

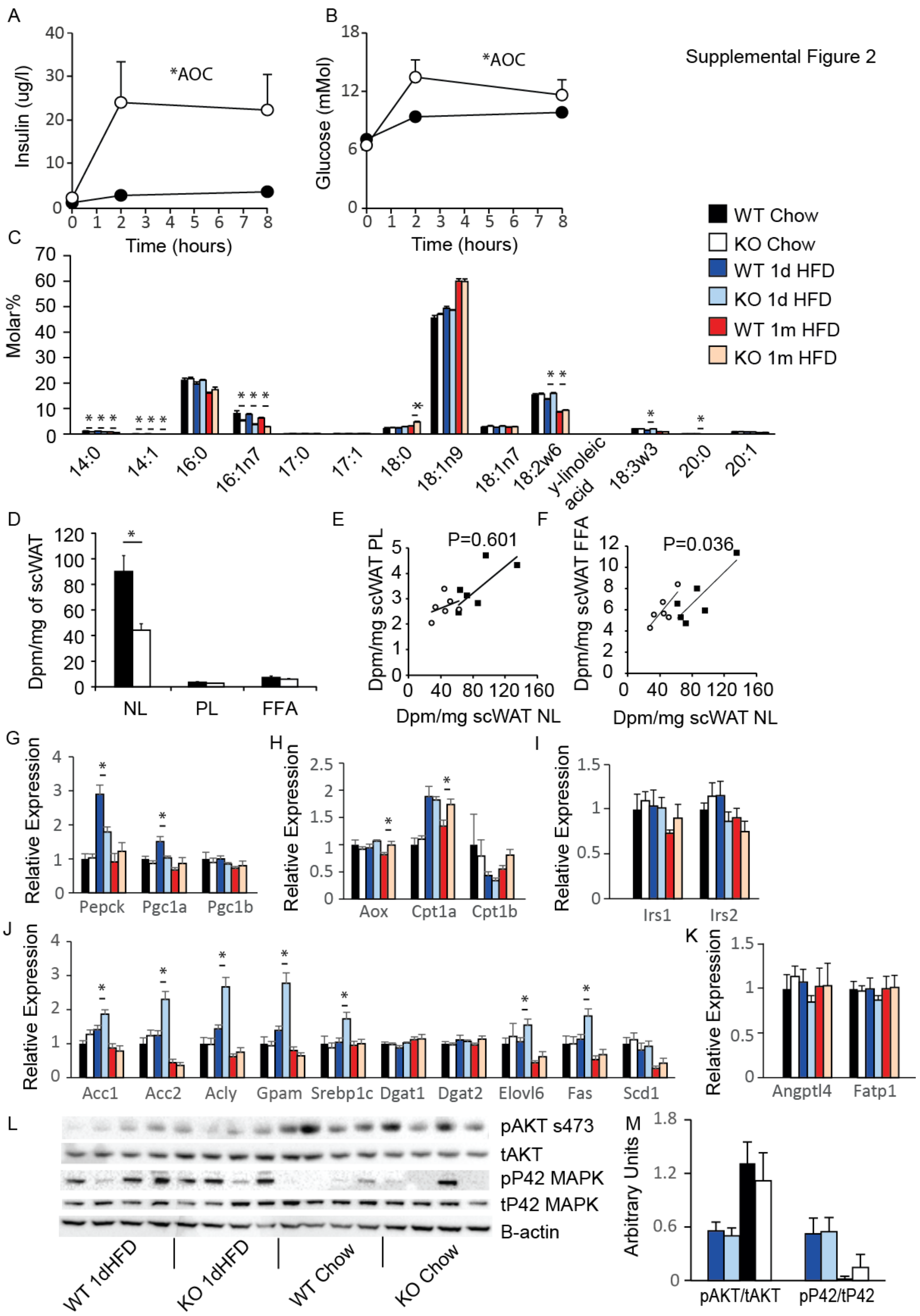

Figure S2 related to figures 2 and 3. A) Serum insulin and B) blood glucose for WT and PPARγ2 KO mice refed a T=0 following an overnight fast. N= 10 WT and 6 KO mice. C) Fatty acid methyl ester analysis by GC-MS for WT and PPARγ2 KO mice. N= 8 per group KO Chow, 1day HFD WT and KO, N=7 per group, WT Chow, 1month HFD WT and KO. D) Palmitate uptake and incorporation into different lipid fractions during a euglycaemic hyperinsulinaemic clamp in subcutaneous white adipose tissues expressed as absolute levels per mg of tissue. Relationships between palmitate incorporation into different fractions showing E) Phospholipid (PL) against neutral lipid (NL) F) Free fatty acids (FFA) against NL N =6 per group. Gene expression analysed by real-time PCR in the liver of chow, 1dHFD or 1 month high-fat fed wild type and PPARγ2 KO mice for: G) Gluconeogenic markers H) Fatty acid oxidative markers I) Insulin sensitivity markers J) lipid biosynthetic genes K) lipid uptake genes N=8 per group except 1 month HFD, N=6. Western blots of protein isolated from the Liver of PPARγ2 KO mice; L) representative western blots and M) quantification. N=8 mice per group for pAKT/tAKT, n=4 per group for pP42/tP42. For multiple time points 2-way ANOVA was performed, if significant pairwise comparisons were performed by two-tailed students T-test, *P<0.05. For determining differences in relationships between NL and PL and FFAs and ANCOVA model was used and reported p-values are for genotype effects. *P<0.05. All data represented as mean ±SEM.

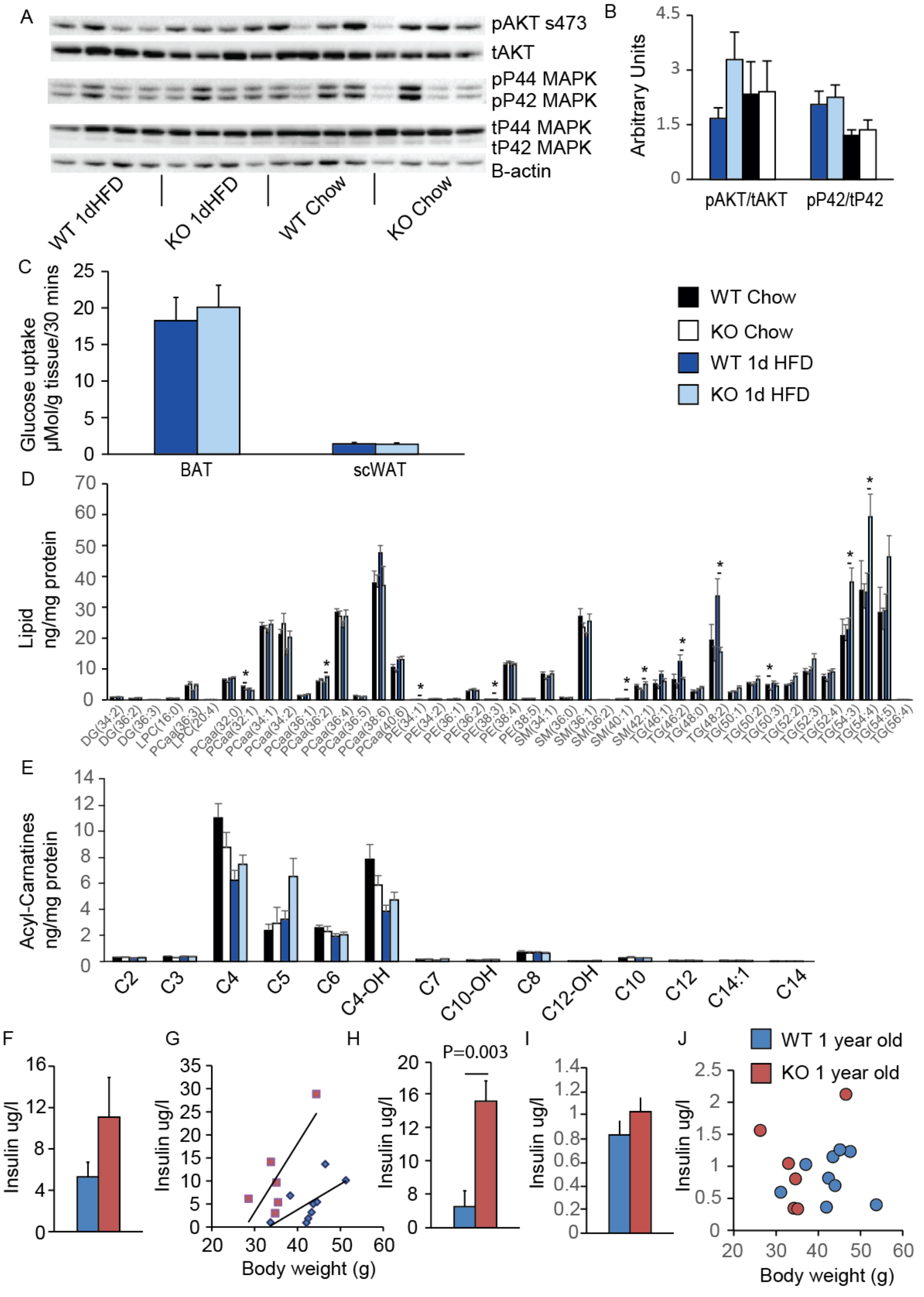

Supplemental Figure 3

Figure S3 related to figures 3 and 4. Western blots of protein isolated from the BAT of PPARγ2 KO mice; A) representative western blots and B) quantification. N=8 mice per group. C) Glucose uptake into BAT and scWAT of WT and PPARγ2 KO mice following 1dHFD. N=9 WT and 7 KO mice per group, 3 months old. Lipid profiles of skeletal muscle from WT and PPARγ2 KO mice fed either chow or 1dHFD showing D) neutral and phospholipids analysed by LC-MS E) acyl-carnatines analysed by tandem MS. N=6-8 per group *P<0.05, students T-test. All mice 4-5 months old. Serum insulin from 1 year old WT and PPARγ2 KO mice F) Raw values for fed insulin G) scatter plot of fed insulin against body weight showing a positive correlation H) Fed insulin levels corrected for body weight with ANCOVA. I) Raw values for fasted insulin J) Scatter plot of fasted insulin against body weight showing no correlation. N= 9 WT and N=6 KO, male mice, chow fed. Raw insulin values analysed by two-tailed students T-test. If body weight showed a significant correlation with insulin concentration in an ANCOVA model, estimated marginal means were reported. *P<0.05. All data represented as mean ±SEM.

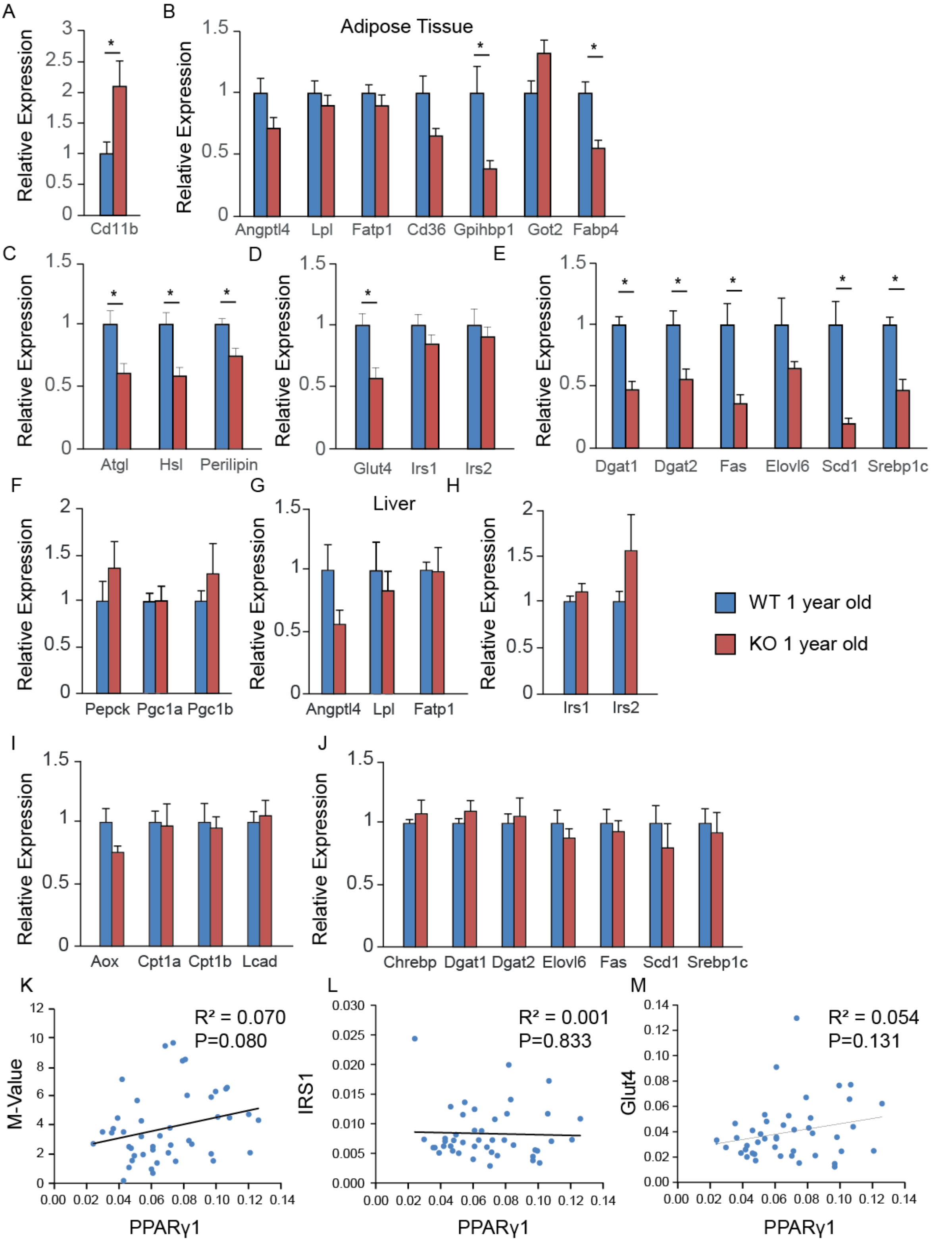

Supplemental Figure 4

Figure S4 related to figure 4. Gene expression analysed by real-time PCR from PPARγ2 KO and WT mice aged to 1 year from adipose tissue A) Cd11b, B) Lipid uptake genes, C) lipolysis markers, D) insulin sensitivity markers and E) lipid biosynthetic genes. Gene expression from PPARγ2 KO and WT mice aged to 1 year from liver for F) Gluconeogenic markers G) Lipid uptake genes H) Insulin sensitivity markers I) Fatty acid oxidative genes J) Lipid biosynthetic and storage genes. N=10 WT, N=6 KO. *$P<0.05$ two-tailed students T-test. Correlations between PPARγ1 expression in human scWAT and K) M-Value during a clamp L) IRS1 expression and M) Glut 4 expression. N=45 subjects. Correlations are Pearson's, exact P-values are reported. All data represented as mean ±SEM.

Table S1. Real time PCR primer sequences relating to STAR methods

Gene code	Species	For	Rev	Probe
18S	Various	CGGCTACCACATCCAAGGAA	GTCGGAATTACCGCGGCT	GAGGGCAAGTCTGGTGCCAG
36B4	Mus musculus	AGATGCAGCAGATCCGCAT	GTTCTTGCCCATCAGCACC	
ACC1	Mus musculus	TGCAGATGACTTCCCTAACCTCTT	CCAGATGTCCGAGATTGTTTTGC	
ACC2	Mus musculus	CGAGGCCACGTCATTGC	CTGAAGTAACCCCACACGTTCTT	
ACLY	Mus musculus	TCACACTGCCAACTTCCTCCTTA	ACTCAGAAAAAGATGCTGTCCTACTG	
ALPHA ENOLASE	Mus musculus	CCCTCTTTCCTTGCTTTGCA	TGGCGTGGATCCTGAGAATAG	
ANGPTL4	Mus musculus	CAAGATGACCCAGCTCATTGG	ATCACTGTCCAGCCTCCATC	
AOX	Mus musculus	AATTGGCACCTACGCCCAG	AGTGGTTTCCAAGCCTCGAA	
AP2 (FABP4)	Mus musculus	CACCGCAGACGACAGGAAG	GCACCTGCACCAGGGC	TGAAGAGCATCATAACCCTAGATGGCGG
ATGL	Mus musculus	CGCCTCTCGAAGGCTCTCT	TGTAGCCCTGTTTGCACATCTC	
CAC	Mus musculus	GCATGTGCCTGGTGTTTGTG	CAGACAAACTTGGTGGTTGTGTCT	
CD11B	Mus musculus	CAGACAGGAAGTAGCAGCTCCT	CTGGTCATGTTGATGAAGGTGCT	
CD36	Mus musculus	GCCAAGCTATTGCGACATGA	TCTCAATGTCCGAGACTTTTCA	CACAGACGCAGCCTCCTTTCC
CHREBPA	Mus musculus	AGTGCTTGAGCCTGGCCTAC	TTGTTCAGGCGGATCTTGTC	
CPT1A	Mus musculus	CCTGGGCATGATTGCAAAG	GCCACTCACGATGTTCTTCGT	
CPT1B	Mus musculus	GCGTGCCAGCCACAATTC	TCCATGCGGTAATATGCTTCAT	CCGGTACTTGGATTCTGTGCGGCC
DGAT1	Mus musculus	AGGTTCTCTAAAAATAACCTTGCATT	TCGTGGTATCCTGAATTGGTG	
DGAT2	Mus musculus	GGCGCTACTTCCGAGACTAC	TGGTCAGCAGGTTGTGTGTC	
ELOVL6	Mus musculus	TGCAGGAAAACTGGAAGAAGTCT	ATGCCGACCACCAAAGATAAA	
F4/80	Mus musculus	CAGATACAGCAATGCCAAGCA	GATTGTGAAGGTAGCATTCACAAGTG	6TGCAGGGCAGGGATCTTGGTTATGC0
FAS	Mus musculus	GCCCAGACAGAGAAGAGGCA	CTGACTCGGGCAACTTCCC	6GGAGGAGGTGGTGATAGCCGGTATGTC0
FATP1	Mus musculus	CGTTTCGATGGTTATGTTAGTGACA	CATCACTAGCACGTCACCTGAGA	
GAPDHC	Mus musculus	AAGGTGGCAGAGGCCTTTG	TGCCCATTTAGCATCTCCTTCT	
GAPDHM	Mus musculus	AGCTGGTAGGCGCCATTG	GGGCGATGGCGAGGTT	
GLUT1	Mus musculus	GCTTCCTGCTCATCAATCGTAAC	CATCGGCTGTCCCTCGAA	
GLUT4	Mus musculus	ACTCATTCTTGGACGGTTCCTC	CACCCCGAAGATGAGTGGG	TGGCGCCTACTCAGGGCTAACATCA
GOT2	Mus musculus	TGGAGTCACAGCTGAAGATCTTG	CACCTCTTGCAACCATTGCTT	
GPAM	Mus musculus	CAATGGCGTACTTCATGTGTTCA	GCACCTCTTATTCAGGACTGCAT	
GPIHBP1	Mus musculus	CAGCAAACCCTTCTGCATCA	ACCACATGGAGTAGGTAGTCAGGTAA	
HSL	Mus musculus	GGAGCACTACAAACGCAACGA	TCGGCCACCGGTAAAGAG	CAGGCCTCAGTGTGACCGCCAGTT
IRS1	Mus musculus	TCCAGAAGCAGCCAGAGGA	AGGATTTGCTGAGGTCATTTAGGT	CAATAGCGTAACTGGACATCACAGCAGAATGAA
IRS2	Mus musculus	GCCTACACGCCTATCGCTAGA	CTCTTGGGCTCTGTGGGTAGA	CAGACCCTTCCCAGCATGCAAGAGTAC
LCAD	Mus musculus	GCATGAAACCAAACGTCTGGA	TGTTTTGTAATTCAGATGCCCAGT	TCCGGTTCTGCTTCCATGGCAAAA
LDH-H	Mus musculus	GCATGTGCCATCAGCATTCT	AGCTTGTCTTCCAACACATCCA	
LDH-M	Mus musculus	GCTGGGTCCTGGGAGAACAT	GACGCCGGCAACATTCAC	
LPL	Mus musculus	TGGAGAAGCCATCCGTGTG	TCATGCGAGCACTTCACCAG	TGCAGAGAGAGGACTCGGAGACGTGG
MCD	Mus musculus	CGGGACCTTCCTCATAAAGAGA	GATAGGCGACAGGCTTGAAAA	
PEPCK	Mus musculus	TGTGGGCGATGACATTGC	TGGCATTTGGATTTGTCTTCAC	6TATCAACCCAGAAAACGGGTTTTTTG0
PERILIPIN	Mus musculus	TGGACCACCTGGAGGAAAAG	TTCGAAGGCGGGTAGAGATG	
PFK-L	Mus musculus	GCCACCATCAGCAACAATGT	GACTGCTTGATGCGATCACAA	
PFK-M	Mus musculus	GGAGATCGTAGACGCCATCAC	CGGCCCATCACTTCTAACACA	
PFK-P	Mus musculus	CCTTCGTCCTGGAGGTGATG	CGGCACCGCAAGTCAAG	
PGC1A	Mus musculus	AACCACACCCACAGGATCAGA	CTCTTCGCTTTATTGCTCCATGA	6CAAACCCTGCCATTGTTAAGACCGAGAA0
PGC1B	Mus musculus	GGCCTTGTGTCAAGGTGGAT	GGTGCTTATGCAGTTCCGTACA	AGACCCCCACACTGCGGGCTC
PPARG1	Mus musculus	TTTAAAAACAAGACTACCCTTTACTGAAATT	AGAGGTCCACAGAGCTGATTCC	6AGAGATGCCATTCTGGCCCCACCAACTT0
PPARG2	Mus musculus	GATGCACTGCCTATGAGCACTT	AGAGGTCCACAGAGCTGATTCC	6AGAGATGCCATTCTGGCCCCACCAACTT0
SCD1	Mus musculus	CTTGCGGATCTTCCTTATCATT	GATCTCGGGCCCATTCG	6ACCATGGCGTTCCAGAATGACGTGT0
SREBP1C	Mus musculus	GCCATGGATTGCACATTTGA	GGCCCGGGAAGTCACTG	GACATGCTCCAGCTCATCAACAACCAAG
PPARY1	Human	GGCCGCAGATTTGAAAGAAG	GGAGAGATCCACGGAGCTGAT	
PPARY2	Human	AAACCCCTATTCCATGCTGTTATG	TGTCAACCATGGTCATTTCTTGTG	
