# Supplementary material for: Peroxisome Proliferator-Activated Receptor γ2 Controls the Rate of Adipose Tissue Lipid Storage and Determines Metabolic Flexibility
Source: Cell Rep. 2018 Aug 24;24(8):2005–2012.e7. doi: 10.1016/j.celrep.2018.07.063 (PMC6113930; doi:10.1016/j.celrep.2018.07.063)
Supplement: Document S2. Article plus Supplemental Information [file mmc2.pdf]

Cell Reports

Report

Peroxisome Proliferator-Activated Receptor γ2 Controls the Rate of Adipose Tissue Lipid Storage and Determines Metabolic Flexibility

Graphical Abstract

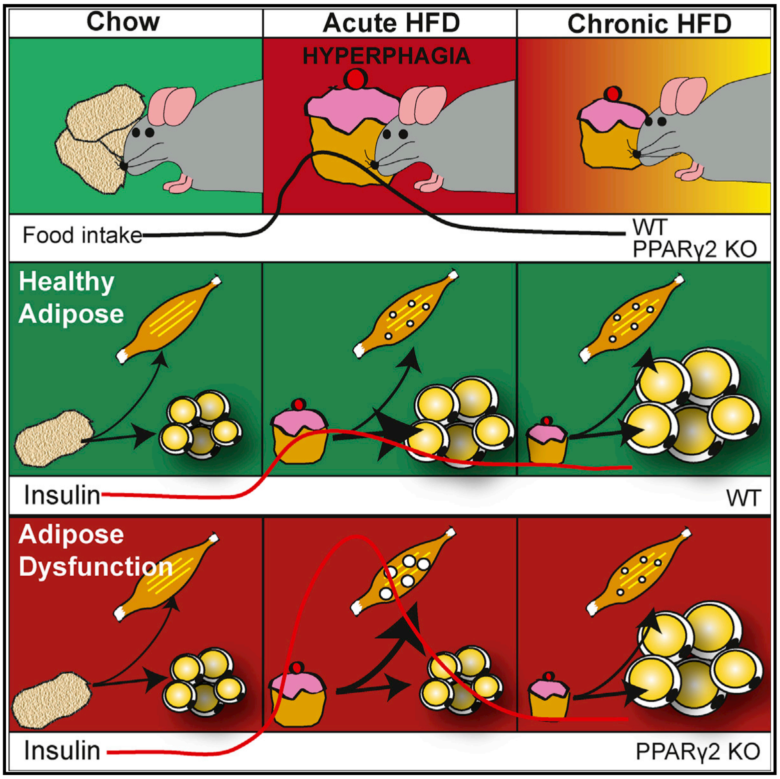

Authors

Sam Virtue, Kasparas Petkevicius, José Maria Moreno-Navarrete, ..., Albert Koulman, José Manuel Fernández-Real, Antonio Vidal-Puig

Correspondence

sv234@medschl.cam.ac.uk (S.V.), ajv22@medschl.cam.ac.uk (A.V.-P.)

In Brief

Virtue et al. describe how the transcription factor peroxisome proliferator-activated receptor gamma 2 (PPARγ2) regulates lipid storage rate in adipose tissue. Mice lacking PPARγ2 cope when fat storage demands are low, but acute overfeeding overwhelms adipose tissue, redirecting lipid to muscle and causing insulin resistance.

Highlights

- Mice lacking PPARγ2 have impaired adipose tissue lipid storage rate
- Low adipose tissue storage rate leads to metabolic inflexibility
- Acute hypercaloric challenges can detect impaired adipose tissue lipid storage rate
- Chronic adipose tissue metabolic inflexibility leads to insulin resistance with age

Virtue et al., 2018, Cell Reports *24*, 2005–2012
August 21, 2018 © 2018 The Authors.
https://doi.org/10.1016/j.celrep.2018.07.063

CellPress

Cell Reports
Report

OPEN ACCESS
CellPress

Peroxisome Proliferator-Activated Receptor γ2 Controls the Rate of Adipose Tissue Lipid Storage and Determines Metabolic Flexibility

Sam Virtue,[1,4,*] Kasparas Petkevicius,[1] José Maria Moreno-Navarrete,[2] Benjamin Jenkins,[1] Daniel Hart,[1] Martin Dale,[1] Albert Koulman,[1] José Manuel Fernández-Real,[2] and Antonio Vidal-Puig[1,3,*]
[1]The University of Cambridge Metabolic Research Laboratories, Wellcome Trust–MRC Institute of Metabolic Science, Cambridge CB2 0QQ, UK
[2]Biomedical Research Institute of Girona (IDIBGI), CIBERobn Pathophysiology of Obesity and Nutrition, Hospital of Girona "Dr. Josep Trueta," Avinguda de França s/n, and Department of Medical Sciences, Faculty of Medicine, University of Girona, Girona, Spain
[3]Wellcome Trust Sanger Institute, Hinxton CB10 1SA, UK
[4]Lead Contact
*Correspondence: sv234@medschl.cam.ac.uk (S.V.), ajv22@medschl.cam.ac.uk (A.V.-P.)
https://doi.org/10.1016/j.celrep.2018.07.063

SUMMARY

One understudied function of white adipose tissue (AT) is its role in postprandial lipid buffering. In this study, we demonstrate that mice lacking the adipose tissue-specific transcription factor peroxisome proliferator-activated receptor γ2 (PPARγ2) exhibit a defect in their rate of adipose tissue lipid storage. Impaired adipose tissue storage rate reduces metabolic flexibility, without compromising fasted glucose tolerance or insulin sensitivity, even following prolonged high-fat feeding. However, acutely overfeeding PPARγ2-KO mice caused a 10-fold increase in insulin levels compared with controls. Although impaired adipose tissue storage rate did not result in insulin resistance in young mice, 1-year-old PPARγ2-KO mice developed skeletal muscle insulin resistance. Our data indicate that failed adipose tissue storage may occur prior to defects in glucose handling and that overfeeding protocols may uncover genes controlling adipose tissue storage rate, as opposed to capacity, and act as a diagnostic test for early-stage human metabolic disease.

INTRODUCTION

The epidemic of obesity has led to epidemics of diabetes and cardiovascular disease. An important question is how obesity leads to metabolic complications. A proposed mechanism linking obesity to metabolic complications is the concept of adipose tissue (AT) expansion and lipotoxicity. The adipose tissue expansion hypothesis suggests that metabolic complications occur when an individual's adipose tissue storage capacity is exceeded (Virtue and Vidal-Puig, 2010), that preventing adipose tissue expansion will worsen metabolic complications (Danforth, 2000), whereas unlimited adipose tissue expansion would be metabolically protective, a concept demonstrated in mice (Kim et al., 2007).

We previously reported (Medina-Gomez et al., 2005) the phenotype of mice lacking peroxisome proliferator-activated receptor γ2 (PPARγ2). PPARγ2 is the adipose tissue-specific isoform of PPARγ. Surprisingly, despite defective adipogenesis *in vitro*, mice lacking PPARγ2 had normal adipocyte size on a chow diet (Medina-Gomez et al., 2005), as well as normal glucose and insulin tolerance on chow or chronic high-fat (3 months of feeding) diets. However, crossing the PPARγ2-knockout (KO) mouse onto an ob/ob background (POKO mouse) led to severe metabolic syndrome. Importantly, defects in insulin sensitivity in POKO mice occurred before any divergence in adiposity between genotypes (Medina-Gomez et al., 2007). The early onset of metabolic impairments in POKO mice raised the question as to which aspect of leptin deficiency was driving the phenotypic divergence. Furthermore, why PPARγ2-KO mice on a non-obese background were metabolically healthy was unresolved.

Although PPARy2 KO mice had normal-sized adipocytes on a chow diet, they showed molecular markers of impaired adipocyte function (Medina-Gomez et al., 2005). Dysfunctional adipose tissue function has been suggested to lead to impaired metabolic flexibility in both humans (Frayn, 2002) and rodents (Asterholm et al., 2012; Asterholm and Scherer, 2010). Metabolic inflexibility is the inability to switch efficiently between predominantly using lipid in the fasted state to carbohydrate in the fed state (Goodpaster and Sparks, 2017; Storlien et al., 2004). In humans, obesity is characterized by low metabolic flexibility (Storlien et al., 2004). Although direct defects in muscle lipid and glucose use have been suggested to cause metabolic inflexibility (Ukropcova et al., 2007), defects in adipose tissue function can also cause metabolic inflexibility by altering the fluxes of lipids to muscle (Frayn, 2002). Dysfunctional adipose tissue fails to store postprandial lipid and redirects it to non-adipose organs. Conversely, in the fasting state, insufficient release of free fatty acids (FFAs) by adipose tissue occurs because of impaired lipolysis (McQuaid et al., 2011).

Although multiple studies have investigated metabolic flexibility in mice (Asterholm et al., 2012; Asterholm and Scherer, 2010; Gao et al., 2015; Isken et al., 2010; Jackowski and Leonardi, 2014; Longo et al., 2008; Muoio et al., 2012; Riachi

This is an open access article under the CC BY license (http://creativecommons.org/licenses/by/4.0/).

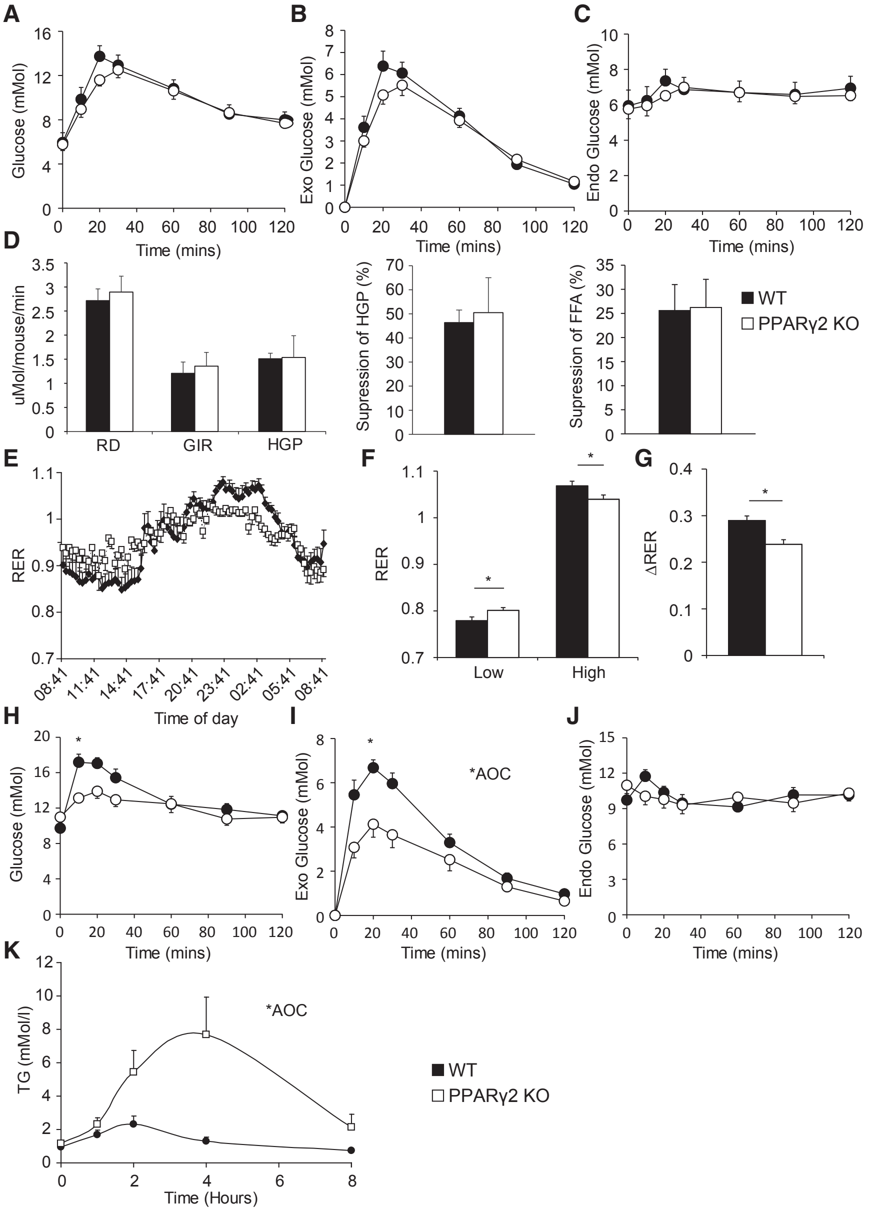

Figure 1. PPARγ2-KO Mice Exhibit Altered Carbohydrate and Lipid Metabolism in the Fed State

(A–C) Fasted glucose tolerance tests from WT and PPARγ2-KO mice: (A) total, (B) exogenous, and (C) endogenous glucose.

(D) Hyperinsulinemic-euglycemic clamps: rate of disposal (RD), glucose infusion rate (GIR), and hepatic glucose production (HGP) in the hyperinsulinemic state (left), suppression of HGP (middle), and suppression of NEFA from basal to hyperinsulinemic state (right) (n = 7 WT and n = 9 KO for GTT, n = 8 WT and n = 5 KO for clamps).

(E–G) Metabolic flexibility shown by (E) representative plot of 24 hr respiratory exchange ratio (RER) determined by indirect calorimetry, (F) lowest and highest 10% of RER values for WT and PPARγ2-KO mice, and (G) dRER (n = 8 mice per group).

(H–J) Fed glucose tolerance tests: (H) total, (I) exogenous, and (J) endogenous glucose (n = 6 WT and n = 9 KO).

(K) Lipid clearance in PPARγ2-KO and WT mice (n = 9 WT, n = 8 KO).

*$p < 0.05$, two-tailed Student's t test. All mice were 4–5 months of age. All data are represented as mean ± SEM. See also Figure S1.

et al., 2004; Vacanti et al., 2014; Vaitheesvaran et al., 2010; Vroegrijk et al., 2013), translating murine data to humans is complicated because of their different feeding patterns. Mice eat multiple small meals per day (Ellacott et al., 2010), and as a result, the rate at which the adipose tissue of mice is required to store and release lipid maybe relatively low. In contrast, humans tend to eat three large meals a day, causing fewer more acute demands for adipose tissue lipid storage (McQuaid et al., 2011). Overall, a loss of adipose tissue flexibility may manifest differently in rodents and humans.

The most commonly used model for inducing obesity and insulin resistance in mice is high-fat diet (HFD) feeding. HFD causes many mouse strains to consume more calories and gain weight, but after an initial period of rapid body weight gain, usually lasting less than 1 week, body weight divergence between HFD and chow-fed mice tends to be relatively slow. The slow rate of weight gain caused by established HFD suggests that chronic HFD may not actually greatly increase demands on adipose tissue storage rate.

In this study, we reinvestigated PPARγ2-KO mice. Although PPARγ is traditionally viewed as a regulator of adipogenesis, PPARγ2 is regulated by fasting and refeeding (Vidal-Puig et al., 1997), suggesting an additional dynamic role for PPARγ2. Supportive of this concept, PPARγ2 is necessary for appropriate expression of lipolytic genes in adipocytes (Rodriguez-Cuenca et al., 2012). In this study, we demonstrate that PPARγ2-KO mice have a reduced adipose tissue lipid storage rate and exhibit metabolic inflexibility. In states in which adipose tissue lipid storage rates are low, mice lacking PPARγ2 have normal insulin and glucose levels. To challenge adipose tissue storage rate, we exploited the fact that mice dramatically over-eat when initially switched to an HFD. In response to 1 day of high-fat feeding (1dHFD), PPARγ2-KO mice failed to store lipid in adipose tissue and redirect it to muscle, causing triglyceride (TG) accumulation and insulin resistance, with PPARγ2-KO mice exhibiting 10 times the insulin levels of controls. Importantly, mice lacking PPARγ2 developed insulin resistance with aging, suggesting that a primary loss of adipose tissue lipid buffering may be initially tolerated but

OPEN ACCESS
CellPress

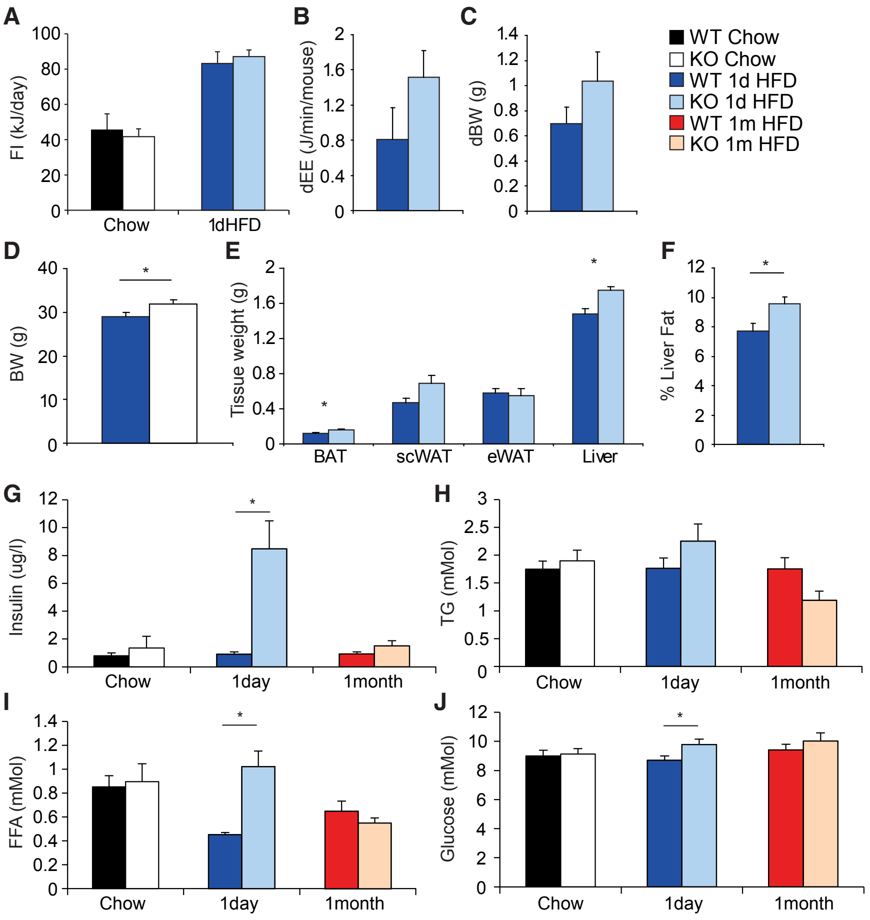

Figure 2. PPARγ2-KO Mice Exhibited Impaired Metabolic Responses to Acute Overfeeding

(A–F) Responses to 1 day high-fat diet (1dHFD): (A) food intake, (B) change in energy expenditure (dEE), and (C) change in body weight (dBW) between chow and after 1dHFD; (D) body weight, (E) tissue weights, and (F) liver fat percentage after 1dHFD period.

(G–J) Serum biochemistry for (G) insulin, (H) TGs, (I) FFAs, and (J) glucose after 1dHFD.

N = 8 per group except 1 month HFD KO and food intake for chow and 1dHFD WT (n = 6). $^{*}p < 0.05$, two-tailed Student's t test. All mice were 4 months of age. All data are represented as mean ± SEM. See also Figures S1 and S2.

ultimately leads to metabolic disease. Finally, we demonstrate that, in line with a potential acute metabolic role for PPARγ2 in adipocytes, PPARγ2 but not PPARγ1 correlates with whole-body and adipose tissue insulin sensitivity in humans.

RESULTS AND DISCUSSION

PPARγ2-KO mice had normal fasting glucose tolerance (Figures 1A–1C) (Medina-Gomez et al., 2005), insulin levels (Figure S1A), glucose infusion rate (GIR), rate of whole-body glucose disappearance (RD), and hepatic glucose production (HGP) during a euglycemic-hyperinsulinemic clamp (Figure 1D). However, PPARγ2-KO mice had reduced metabolic flexibility in the fed state (Figures 1E–1G). On the basis of their amplitude of respiratory exchange ratio (RER), PPARγ2-KO mice had increased glucose oxidation in the light phase and increased lipid oxidation in the dark phase relative to controls (Figure 1F). To investigate the increased glucose oxidation in the light phase, we performed a glucose tolerance test (GTT) in the fed state at 10 a.m., incorporating stable isotopes. Consistent with their RER profile, mice lacking PPARγ2 exhibited increased clearance of exogenous glucose (Figures 1H–1J). The PPARγ2-KO mice also exhibited reduced RER during the night phase, suggesting that PPARγ2 mice may have impaired adipose tissue lipid storage leading to excess delivery of lipid to muscle (Randle et al., 1963), and in line with this concept, mice lacking PPARγ2 were unable to clear an oral lipid load as effectively as controls, exhibiting higher TG (Figure 1K) and FFAs (Figure S1B) during a lipid tolerance test.

In response to chronic HFD, mice lacking PPARγ2 have normal carbohydrate metabolism and adipose tissue mass (Medina-Gomez et al., 2005). However, given their compressed RER profiles and inability to clear lipids, we hypothesized that PPARγ2-KO mice may exhibit a defect in the rate of adipose tissue lipid storage, rather than total storage capacity. This hypothesis was also supported by the early onset (4 weeks of age) of hyperinsulinemia in POKO mice, an age at which ob/ob mice are already hyperphagic but prior to a divergence in adiposity relative to controls (Medina-Gomez et al., 2007). To stress adipose tissue storage rate, we developed an overfeeding challenge by giving mice a high-fat diet for 1 day (1dHFD). We first characterized 1dHFD in wild-type mice. Mice on 1dHFD doubled food intake (Figure S1C) and increased insulin levels (Figure S1D) to maintain normoglycemia (Figure S1E). Body weight and subcutaneous white adipose tissue (scWAT) mass were increased (Figures S1F–S1H). Finally, *Pparγ2* expression in scWAT was physiologically increased in response to 1dHFD, suggestive of a role for PPARγ2 in responding to overfeeding (Figure S1I).

PPARγ2-KO mice placed on HFD for 1 day exhibited similar food intake (Figure 2A), diet-induced thermogenesis (Figure 2B), and body weight gain (Figure 2C) to controls, whereas absolute body weight, BAT weight, liver weight, and liver fat percentage were increased (Figures 2D–2F).

When subjected to 1dHFD, PPARγ2-KO mice exhibited dramatically increased insulin levels and increased glucose and FFA levels compared with 1dHFD wild-type controls. Importantly, prolonged HFD feeding (for either 1 week or 1 month) normalized the serum biochemistry parameters of PPARγ2-KO mice (Figures 2G–2J, S1L, and S1M), even though PPARγ2-KO mice after 1 month of HFD had greater adiposity (Figures S1J

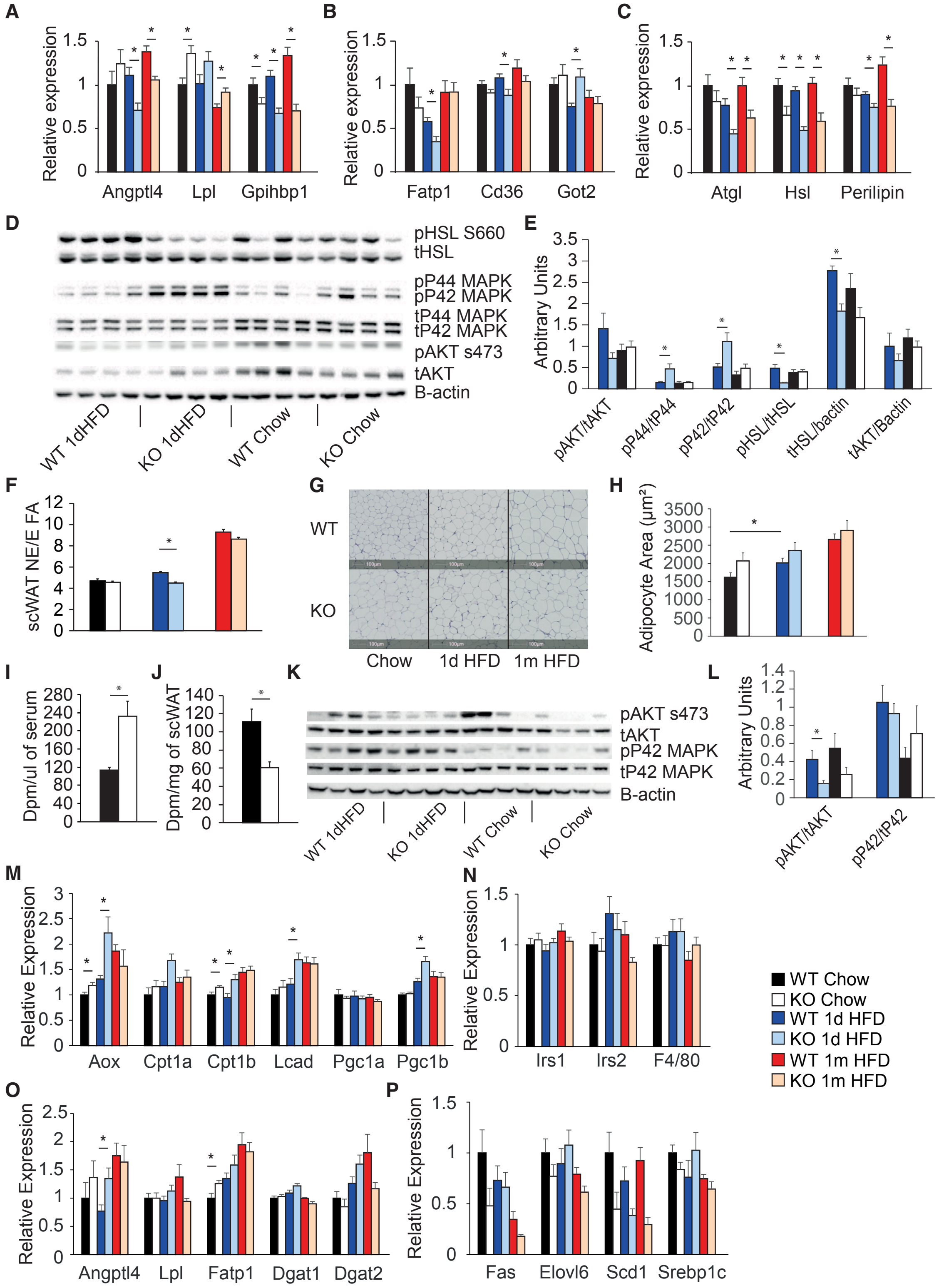

Figure 3. Mice Lacking PPARγ2 Cannot Appropriately Store Lipid in Adipose Tissue

(A–C) Gene expression in WAT for (A) lipoprotein lipase and regulatory molecules and (B) FA uptake genes. (C) Markers of lipolysis (n = 8 mice per group except 1 month HFD KO [n = 7]).

(D and E) Western blots of WAT: (D) representative western blots and (E) quantification (n = 8 mice per group).

(legend continued on next page)

and S1K) than PPARγ2-KO mice after 1dHFD (Figure 2E). Finally, we conducted a fasting-refeeding experiment and demonstrated that mice lacking PPARγ2 had greatly increased insulin in response to refeeding after an overnight fast (Figures S2A and S2B). Overall, our data suggested a lack of PPARγ2 impaired adipose tissue lipid storage rate and a failure in adipose tissue storage rate caused insulin resistance in response to overnutrition.

We next sought to confirm that PPARγ2-KO adipose tissue was unable to buffer lipid appropriately. We first analyzed gene expression in scWAT. In terms of genes controlling hydrolysis of TG to FFAs, lipoprotein lipase (*Lpl)* tended to be upregulated, whereas the LPL inhibitory protein angiopoietin-like 4 (*Angptl4)* was downregulated in PPARγ2-KO scWAT after 1dHFD (Figure 3A). The FFA transporter-encoding genes FA transport protein (*Fatp1)* and *Cd36* were downregulated in PPARγ2-KO mice by 1dHFD (Figure 3B). The trend to upregulation of *Lpl* and the downregulation of *Angptl4*, *Cd36*, and *Fatp1* were consistent with the normal TG levels and increased FFAs in PPARγ2-KO mice following 1dHFD. To exclude a role for lipolysis in regulating circulating FFA levels, we analyzed expression of adipose TG lipase (*Atgl),* which was downregulated under high-fat feeding conditions and hormone sensitive lipase (*Hsl*), which was downregulated under all nutritional conditions (Figure 3C) in PPARγ2-KO mice. HSL phosphorylation at the activatory S660 site was decreased in the scWAT of PPARγ2-KO mice following 1dHFD (Figures 3D and 3E), while insulin signaling, a known suppressor of lipolysis, was either similar (ratio of phospho-AKT to total AKT) or upregulated (ratio of phospho-P44/42 ERK to total P44/42 ERK), in line with the increased circulating insulin levels. Overall, and in line with our previous work (Rodriguez-Cuenca et al., 2012), the elevated FFAs in PPARγ2-KO mice were unlikely to be a result of FFA release by adipose tissue but a result of a defect in FFA uptake.

To confirm a defect in FFA transport into adipose tissue, we used the fact our HFD has an increased nonessential (NE)/essential (E) FA ratio compared with chow. Feeding mice an HFD leads to a progressive increase in NE/E FA ratio in white adipose tissue (WAT) (Yew Tan et al., 2015). Wild-type (WT) and PPARγ2-KO mice had similar NE/E FA ratios under chow conditions, but 1 day HFD increased NE/E FA ratio only in WT mice (Figures 3F and S2C), a difference that was normalized between genotypes after 1 month of HFD. Importantly, this analysis detects existing fat as well as newly stored fat. The 15% increase in NE/E ratio in WT mice was in good agreement with the 20% increase in fat mass observed in response to 1dHFD (Figure S1G), suggesting that most of the fat accumulating in the scWAT of WT mice was dietary derived. Further support for a lack of lipid uptake to adipocytes was provided by histological analysis of WT and PPARγ2-KO mouse adipocyte sizes. Although WT mice increased their adipocyte size following 1dHFD, PPARγ2-KO mice did not (Figures 3G and 3H). Finally, we traced palmitate uptake to WAT under euglycemic-hyperinsulinemic clamp conditions. PPARγ2-KO mice exhibited increased ^{14}C-palmitate in blood (Figure 3I) and decreased incorporation into adipose tissue (Figures 3J and S2D–S2F). Overall, these results suggested that elevated circulating FFA levels in PPARγ2-KO mice were caused by a failure to uptake and esterify FFAs into WAT, a concept further supported by the elevated FFAs during a lipid tolerance test (LTT) (Figure S1B).

The failure of adipose tissue to clear FFAs (Figure 3J) suggested a potential increased flux of FFAs to other organs (Virtue and Vidal-Puig, 2010), which we next sought to investigate. In liver, mice lacking PPARγ2 exhibited a more lipogenic response to acute overfeeding than WTs (Figures S2G–S2K), potentially reflecting the increased insulin levels (Figure 2G), but no differences in FA oxidation markers or changes in the phosphorylation status of insulin signaling molecules (Figures S2L and S2M). Similarly to liver, no defects in brown adipose tissue (BAT) insulin signaling (Figures S3A and S3B), or glucose disposal into BAT or WAT (Figure S3C) were detected. Conversely, AKT phosphorylation was impaired in skeletal muscle after 1dHFD in PPARγ2-KO mice (Figures 3K and 3L). Muscle gene expression showed that several oxidative metabolism genes in PPARγ2-KO mice (Figure 3M) reached levels of expression after 1dHFD that were similar to or greater than those observed for either WT or PPARγ2-KO mice after 1 month of high feeding. Additionally, lipid uptake and TG storage genes tended to be increased in the muscle of PPARγ2-KO mice compared with controls (Figure 3O). HFD did not modify mRNA markers of insulin sensitivity in PPARγ2-KO muscles (Figure 3N) or markers of lipogenesis (Figure 3P) between genotypes.

To validate gene expression changes, we analyzed muscle lipid composition. On a chow diet, WT and PPARγ2-KO mice had largely similar muscle lipidomes (Figure S3D) that could not be separated using multivariate analyses (Figures 4A and 4B), but following 1dHFD, a significant separation in the lipid profiles was detectable (Figures 4A and 4B). PPARy2-KO mice accumulated more longer chain FA-containing TGs than controls (Figure 4C). Finally, we measured the acylcarnitine profile of the muscles. Under chow conditions, and consistent with their increased carbohydrate oxidation in the light phase, mice lacking PPARγ2 had less short-chain acyl carnitines, but this phenotype was reversed by 1dHFD (Figures 4D and S3E). In summary, our data supported the idea that PPARγ2-KO mice exhibit a

(F) The ratio of non-essential to essential FAs in adipose tissue from WT and PPARγ2-KO mice (n = 8 per group KO chow, 1 day HFD WT and KO, n = 7 per group, WT chow, 1 month HFD WT and KO).

(G and H) Morphometric analysis of sections of WAT from WT and PPARγ2-KO mice: (G) representative images (scale bar shows 100 μM) and (H) quantification of adipocyte cross-sectional area (n = 8 per group KO chow, 1 day HFD KO and 1 month HFD WT, n = 7 per group, KO chow, 1dHFD WT 1 month HFD KO).

(I and J) Radioactive palmitate levels in (I) blood and (J) scWAT from WT and PPARγ2-KO mice under hyperinsulinemic-euglycemic clamp conditions (n = 6 per group).

(K and L) Western blots from muscle: (K) representative western blots and (L) quantification (n = 8 per group).

(M–P) Gene expression in muscle for (M) FA oxidative markers, (N) insulin sensitivity markers, (O) lipid uptake and storage genes, and (P) lipid biosynthetic genes (n = 8 mice per group except 1 month HFD KO [n = 7]).

For multiple time points, two-way ANOVA was performed, and if significant, pairwise comparisons were performed using two-tailed Student's t test (*$p < 0.05$). All mice were 4–5 months old. All data are represented as mean ± SEM. See also Figures S2 and S3.

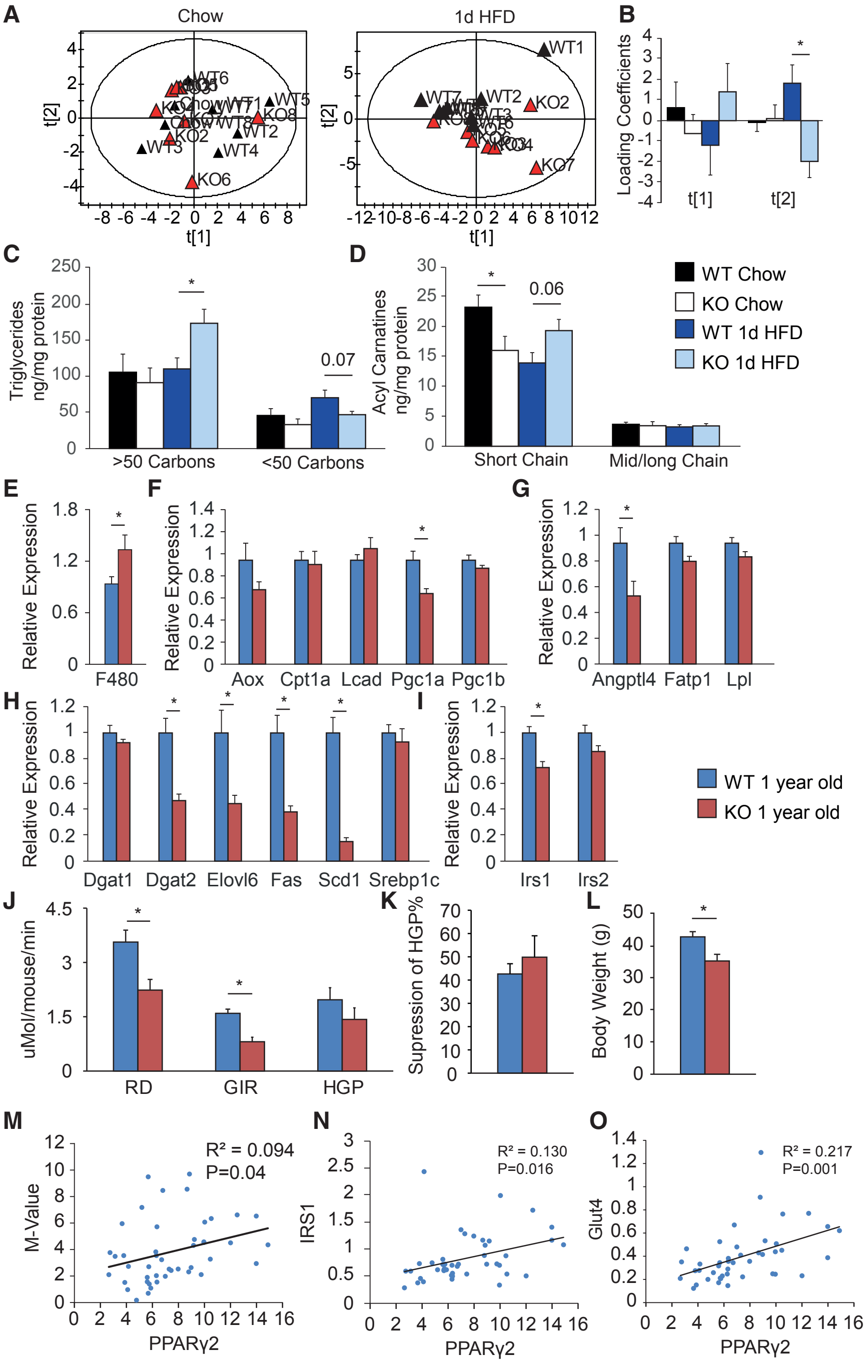

Figure 4. Loss of Adipose Tissue Lipid Buffering Leads to Aging-Related Insulin Resistance

(A) Principal-component analysis of lipid species from muscle of chow (left) or 1dHFD (right) fed mice.

(B) Quantification of the first two principal components from (A).

(C) Muscle TG species.

(D) Muscle acylcarnitines species (n = 6–8 per group).

(E–I) Gene expression in muscle of 1-year-old mice: (E) F4/80, (F) FA oxidative markers, (G) lipid uptake and storage genes, (H) lipid biosynthetic genes, and (I) Insulin sensitivity markers (n = 10 WT, n = 7 KO, male mice chow fed).

(J–L) Clamps from 1-year-old mice: (J) rate of glucose disposal (RD), glucose infusion rate (GIR), and hepatic glucose production (HGP) under hyperinsulinemic condition; (K) suppression of HGP between the basal and hyperinsulinemic state; and (L) body weights of mice used for clamps (n = 7 WT and 6 KO mice, chow fed).

(M–O) Correlations between PPARγ2 expression in human scWAT and (M) M-value during a clamp, (N) IRS1 expression, and (O) Glut 4 expression (n = 45 subjects).

For multiple time points, two-way ANOVA was performed, and if significant, pairwise comparisons were performed using two-tailed Student's t test (*$p < 0.05$). Correlations are Pearson's, and exact p values are reported. All mice were 4–5 months old. All data are represented as mean ± SEM. See also Figures S3 and S4.

primary defect in adipose tissue storage rate, which in the context of acute overfeeding drives FFA flux to muscle. Increased FFA flux to muscle leads to lipid accumulation, lipotoxicity-induced insulin resistance, and compensatory upregulation of the FA oxidation program (Randle et al., 1963).

Although 1dHFD was able to unmask the underlying metabolic defects of PPARγ2 ablation, even under chow conditions, mice lacking PPARγ2 had impaired metabolic flexibility (Figure 1E) and increased expression of the lipid oxidation genes *Aox* and *Cpt1b* (Figure 3M) in skeletal muscle. To determine if a loss of metabolic flexibility due to adipose tissue dysfunction led to longer term metabolic complications, we aged PPARγ2-KO mice and controls on a chow diet for 1 year. Aged mice lacking PPARγ2 exhibited decreased expression of *Pgc1α* (Figure 4F) and insulin sensitivity markers including the FA synthesis program and *Irs1* (Figures 4H and 4I) and increased expression of the macrophage marker *F4/80* (Figure 4E) in muscle. Notably, other than a tendency toward reduced *Scd1* expression, these changes were absent in the muscles of young mice on a chow diet (Figures 3M–3P). These molecular changes manifested in skeletal muscle insulin resistance, with aged PPARγ2-KO mice exhibiting lower RD, reduced GIR, and normal HGP (Figures 4J–4L) as well as increased fed insulin levels (Figures S3F–S3J) compared with WTs. Adipose tissue showed relatively little further impairment with aging compared with young animals (Figures S4A–S4E), and liver exhibited almost no changes between PPARγ2-KO and WTs (Figures S4F–S4J).

The human relevance of our observations was confirmed in adipose tissue from 45 obese subjects who had undergone a

euglycemic-hyperinsulinemic clamp. *PPARγ2*, but not *PPARγ1*, expression in scWAT correlated with M-value as well as *IRS1* and *GLUT4* (Figures 4M–4O and S4K–S4M).

Our study identifies PPARγ2 as a crucial regulator of adipose tissue lipid storage rate, in addition to its role in adipose tissue development and total storage capacity. A reduction in adipose tissue storage rate limits the ability of adipose tissue to respond acutely to large nutrient influxes and causes metabolic inflexibility that chronically leads to insulin resistance. Our data therefore suggest a possible evolutionary rationale for the presence of PPARγ2 in mature adipocytes, as a nutritional integrator of lipid uptake and release that controls the availability of FFAs (Rodriguez-Cuenca et al., 2012) and gluconeogenic substrates (Perry et al., 2015; Titchenell et al., 2016) in the fasted state and permits appropriate lipid storage in the fed state. Because the mouse model of PPARγ2 ablation we have used is a total KO, we cannot exclude effects of PPARγ2 in other organs, but PPARy2 is almost exclusively expressed in adipose tissue (Escher et al., 2001). Furthermore, although it is possible that effects on lipid uptake and release could be secondary to alterations in adipogenesis, we observed these defects in chow-fed mice at an age when PPARγ2-KO animals possess normal adipocyte numbers (Figures 3G and 3H).

Under laboratory conditions of *ad libitum* access to food, mice do not eat like humans, consuming many small meals throughout the day (Ellacott et al., 2010). Conversely, most humans consume a small number of large meals a day and occasionally tend to over-eat. As such, the nutrient load that humans face is more infrequent, with a larger and more variable amplitude than that faced by mice. Although not perfectly analogous to the human breakfast-lunch-dinner feeding pattern, the 1dHFD model in rodents increases the amplitude of their nutrient intake and importantly keeps them in circadian phase. The 1dHFD model may therefore provide a more translatable metabolic challenge than traditional chronic HFDs for the study of adipose lipid storage function, and it is likely that other mouse models with similar defects maybe uncovered using a 1dHFD protocol.

Our results also raise questions regarding how metabolic disease is diagnosed and studied in humans. Currently there is a bias toward studying human and mouse metabolism in the fasted state. Equally, diagnostic tests for human metabolic disease are conducted almost exclusively in the fasted state to control for human meal choices. By extension, our data suggest that there are humans with primary adipose tissue defects who exhibit normal GTT results but become metabolically dysfunctional after eating. It is possible that a hypercaloric mixed meal tolerance test would be the most suitable way to detect such individuals. Finally, our data suggest that “murinizing” human feeding patterns may be a way to treat individuals with adipose dysfunction and storage defects.

STAR★METHODS

Detailed methods are provided in the online version of this paper and include the following:

- KEY RESOURCES TABLE
- CONTACT FOR REAGENT AND RESOURCE SHARING
- EXPERIMENTAL MODEL AND SUBJECT DETAILS
  - Animals
  - Rodent Diets
  - Feeding regimes
- METHOD DETAILS
  - Glucose tolerance test
  - Inlet conditions
  - Temperature Program:
  - MSD conditions:
  - Mouse Euglycaemic-hyperinsulinaemic clamp
  - Mouse 2-Deoxyglucose uptake
  - Calorimetry and assessment of metabolic flexibility
  - Lipid tolerance test
  - Liver fat %
  - Serum Biochemistry
  - RNA extraction and real time PCR
  - Western Blotting
  - Histological analysis of white adipose tissue
  - GC-MS FAME analysis
  - Lipid uptake to adipose tissue
  - LC-MS analaysis
- QUANTIFICATION AND STATISTICAL ANALYSIS
- DATA AND SOFTWARE AVAILABILLITY

SUPPLEMENTAL INFORMATION

Supplemental Information includes four figures and one table and can be found with this article online at https://doi.org/10.1016/j.celrep.2018.07.063.

ACKNOWLEDGMENTS

We thank Helen Westby, Sarah Grocott, Charley Beresford, Jade Bacon, Laura McKinven, and Agnes Lukasik for their excellent technical assistance. All animal work was carried out in the Disease Model Core (MRC Metabolic Diseases Unit [MRC_MC_UU_12012/5]; Wellcome Trust Strategic Award [100574/Z/12/Z]). All serum biochemistry was conducted by the Biochemistry Assay Lab (MRC Metabolic Diseases Unit [MRC_MC_UU_12012/5]). We thank the BHF (RG/12/13/29853), FIS (PI15/01934), and MRC (MC_UU_12012/2) for funding this work.

AUTHOR CONTRIBUTIONS

S.V. designed and conducted experiments and wrote the manuscript. K.P., D.H., B.J., and M.D. conducted experiments. A.K. designed and conducted experiments. J.M.M.-N. and J.M.F.-R. provided and analyzed human adipose gene expression data. A.V.-P. designed experiments and wrote the manuscript.

DECLARATION OF INTERESTS

The authors declare no competing interests.

Received: November 27, 2017
Revised: March 15, 2018
Accepted: July 18, 2018
Published: August 21, 2018

REFERENCES

Asterholm, I.W., and Scherer, P.E. (2010). Enhanced metabolic flexibility associated with elevated adiponectin levels. Am. J. Pathol. *176*, 1364–1376.

Asterholm, I.W., Mundy, D.I., Weng, J., Anderson, R.G., and Scherer, P.E. (2012). Altered mitochondrial function and metabolic inflexibility associated with loss of caveolin-1. Cell Metab. *15*, 171–185.

Danforth, E., Jr. (2000). Failure of adipocyte differentiation causes type II diabetes mellitus? Nat. Genet. *26*, 13.

Ellacott, K.L., Morton, G.J., Woods, S.C., Tso, P., and Schwartz, M.W. (2010). Assessment of feeding behavior in laboratory mice. Cell Metab. *12*, 10–17.

Escher, P., Braissant, O., Basu-Modak, S., Michalik, L., Wahli, W., and Desvergne, B. (2001). Rat PPARs: quantitative analysis in adult rat tissues and regulation in fasting and refeeding. Endocrinology *142*, 4195–4202.

Folch, J., Lees, M., and Sloane Stanley, G.H. (1957). A simple method for the isolation and purification of total lipides from animal tissues. J. Biol. Chem. *226*, 497–509.

Frayn, K.N. (2002). Adipose tissue as a buffer for daily lipid flux. Diabetologia *45*, 1201–1210.

Gao, S., McMillan, R.P., Zhu, Q., Lopaschuk, G.D., Hulver, M.W., and Butler, A.A. (2015). Therapeutic effects of adropin on glucose tolerance and substrate utilization in diet-induced obese mice with insulin resistance. Mol. Metab. *4*, 310–324.

Goodpaster, B.H., and Sparks, L.M. (2017). Metabolic flexibility in health and disease. Cell Metab. *25*, 1027–1036.

Isken, F., Klaus, S., Petzke, K.J., Loddenkemper, C., Pfeiffer, A.F., and Weickert, M.O. (2010). Impairment of fat oxidation under high- vs. low-glycemic index diet occurs before the development of an obese phenotype. Am. J. Physiol. Endocrinol. Metab. *298*, E287–E295.

Jackowski, S., and Leonardi, R. (2014). Deregulated coenzyme A, loss of metabolic flexibility and diabetes. Biochem. Soc. Trans. *42*, 1118–1122.

Kim, J.Y., van de Wall, E., Laplante, M., Azzara, A., Trujillo, M.E., Hofmann, S.M., Schraw, T., Durand, J.L., Li, H., Li, G., et al. (2007). Obesity-associated improvements in metabolic profile through expansion of adipose tissue. J. Clin. Invest. *117*, 2621–2637.

Longo, K.A., Charoenthongtrakul, S., Giuliana, D.J., Govek, E.K., McDonagh, T., Qi, Y., DiStefano, P.S., and Geddes, B.J. (2008). Improved insulin sensitivity and metabolic flexibility in ghrelin receptor knockout mice. Regul. Pept. *150*, 55–61.

McQuaid, S.E., Hodson, L., Neville, M.J., Dennis, A.L., Cheeseman, J., Humphreys, S.M., Ruge, T., Gilbert, M., Fielding, B.A., Frayn, K.N., and Karpe, F. (2011). Downregulation of adipose tissue fatty acid trafficking in obesity: a driver for ectopic fat deposition? Diabetes *60*, 47–55.

Medina-Gomez, G., Virtue, S., Lelliott, C., Boiani, R., Campbell, M., Christodoulides, C., Perrin, C., Jimenez-Linan, M., Blount, M., Dixon, J., et al. (2005). The link between nutritional status and insulin sensitivity is dependent on the adipocyte-specific peroxisome proliferator-activated receptor-gamma2 isoform. Diabetes *54*, 1706–1716.

Medina-Gomez, G., Gray, S.L., Yetukuri, L., Shimomura, K., Virtue, S., Campbell, M., Curtis, R.K., Jimenez-Linan, M., Blount, M., Yeo, G.S., et al. (2007). PPAR gamma 2 prevents lipotoxicity by controlling adipose tissue expandability and peripheral lipid metabolism. PLoS Genet. *3*, e64.

Muoio, D.M., Noland, R.C., Kovalik, J.P., Seiler, S.E., Davies, M.N., DeBalsi, K.L., Ilkayeva, O.R., Stevens, R.D., Kheterpal, I., Zhang, J., et al. (2012). Muscle-specific deletion of carnitine acetyltransferase compromises glucose tolerance and metabolic flexibility. Cell Metab. *15*, 764–777.

Perry, R.J., Camporez, J.G., Kursawe, R., Titchenell, P.M., Zhang, D., Perry, C.J., Jurczak, M.J., Abudukadier, A., Han, M.S., Zhang, X.M., et al. (2015). Hepatic acetyl CoA links adipose tissue inflammation to hepatic insulin resistance and type 2 diabetes. Cell *160*, 745–758.

Randle, P.J., Garland, P.B., Hales, C.N., and Newsholme, E.A. (1963). The glucose fatty-acid cycle. Its role in insulin sensitivity and the metabolic disturbances of diabetes mellitus. Lancet *1*, 785–789.

Riachi, M., Himms-Hagen, J., and Harper, M.E. (2004). Percent relative cumulative frequency analysis in indirect calorimetry: application to studies of transgenic mice. Can. J. Physiol. Pharmacol. *82*, 1075–1083.

Rodriguez-Cuenca, S., Carobbio, S., Velagapudi, V.R., Barbarroja, N., Moreno-Navarrete, J.M., Tinahones, F.J., Fernandez-Real, J.M., Oresic, M., and Vidal-Puig, A. (2012). Peroxisome proliferator-activated receptor γ-dependent regulation of lipolytic nodes and metabolic flexibility. Mol. Cell. Biol. *32*, 1555–1565.

Storlien, L., Oakes, N.D., and Kelley, D.E. (2004). Metabolic flexibility. Proc. Nutr. Soc. *63*, 363–368.

Titchenell, P.M., Quinn, W.J., Lu, M., Chu, Q., Lu, W., Li, C., Chen, H., Monks, B.R., Chen, J., Rabinowitz, J.D., and Birnbaum, M.J. (2016). Direct hepatocyte insulin signaling is required for lipogenesis but is dispensable for the suppression of glucose production. Cell Metab. *23*, 1154–1166.

Ukropcova, B., Sereda, O., de Jonge, L., Bogacka, I., Nguyen, T., Xie, H., Bray, G.A., and Smith, S.R. (2007). Family history of diabetes links impaired substrate switching and reduced mitochondrial content in skeletal muscle. Diabetes *56*, 720–727.

Vacanti, N.M., Divakaruni, A.S., Green, C.R., Parker, S.J., Henry, R.R., Ciaraldi, T.P., Murphy, A.N., and Metallo, C.M. (2014). Regulation of substrate utilization by the mitochondrial pyruvate carrier. Mol. Cell *56*, 425–435.

Vaitheesvaran, B., LeRoith, D., and Kurland, I.J. (2010). MKR mice have increased dynamic glucose disposal despite metabolic inflexibility, and hepatic and peripheral insulin insensitivity. Diabetologia *53*, 2224–2232.

Vidal-Puig, A.J., Considine, R.V., Jimenez-Liñan, M., Werman, A., Pories, W.J., Caro, J.F., and Flier, J.S. (1997). Peroxisome proliferator-activated receptor gene expression in human tissues. Effects of obesity, weight loss, and regulation by insulin and glucocorticoids. J. Clin. Invest. *99*, 2416–2422.

Virtue, S., and Vidal-Puig, A. (2010). Adipose tissue expandability, lipotoxicity and the Metabolic Syndrome–an allostatic perspective. Biochim. Biophys. Acta *1801*, 338–349.

Vroegrijk, I.O., van Diepen, J.A., van den Berg, S.A., Romijn, J.A., Havekes, L.M., van Dijk, K.W., Darland, G., Konda, V., Tripp, M.L., Bland, J.S., and Voshol, P.J. (2013). META060 protects against diet-induced obesity and insulin resistance in a high-fat-diet fed mouse. Nutrition *29*, 276–283.

Yew Tan, C., Virtue, S., Murfitt, S., Roberts, L.D., Phua, Y.H., Dale, M., Griffin, J.L., Tinahones, F., Scherer, P.E., and Vidal-Puig, A. (2015). Adipose tissue fatty acid chain length and mono-unsaturation increases with obesity and insulin resistance. Sci. Rep. *5*, 18366.

STAR★METHODS

KEY RESOURCES TABLE

REAGENT or RESOURCE	SOURCE	IDENTIFIER
Antibodies		
pHSL S660	Cell Signaling	Cat 4126; RRID: AB_490997
Total HSL	Cell Signaling	Cat 4107 ; RRID: AB_2296900
Phospho P44/42 MAPK	Cell Signaling	Cat 4376; RRID: AB_331772
Total P44/42 MAPK	Cell Signaling	Cat 4695; RRID: AB_390779
pAKT S473	Cell Signaling	Cat 4060; RRID: AB_2315049
Total AKT	Cell Signaling	Cat 2920; RRID: AB_1147620
B-actin	Cell Signaling	Cat 8226; RRID: AB_306371
Biological Samples		
Human adipose tissue biopsies	Endocrinology Service of the Hospital of Girona 'Dr Josep Trueta'	N/A
Chemicals, Peptides, and Recombinant Proteins		
D-Glucose (6,6-D2 99%)	Goss Scientific	DLM-349-1
D-Glucose (2-D, 98%)	Goss Scientific	DLM-1271-1
Pyridine	Sigma	270970-100ml
Hydroxyamine hydrochloride	Sigma	431362-50 g
Acetic anhydride	Sigma	320102
Ethyl Acetate	Sigma	34972
Fentanyl	Martindale Pharma	PL 00156/0038
Midazolam (Hypnovel)	Roche	PL00031/0126
Acepromazine	Novartis	GTIN5037694004606
3-3H glucose	Perkin Elmer	NET331A250UC
Deoxy-D-glucose, 2-[1-14C]	Perkin Elmer	NEC495A250UC
Insulin	Novo Nordisk	EU/1/02/230/003
Olive oil	Heritage	
STAT 60	AMS Biotech	CS-502
Reverse transcriptase kit	N/A	N/A
Reverse Transcriptase M-MLV	Promega	M170b
M-MLV RT 5x buffer	Promega	M351A
MgCl2 25mM	Promega	A351B
Random Primers	Promega	C118A
dNTP Mix 10 mM	Promega	U151B
RNeasy Lipid Tissue Mini Kit	QIAGEN	74804
Chlorofom	Sigma	34854
Methanol	Sigma	34860
BF3 Methanol	Sigma	B1127
Hexane	Sigma	34859
1-14C Palmitate	Perkin Elmer	NEC075H050UC
BondElut NH2 columns	Agilent	12102014
Isolute Strong anion exchange columns, 100 mg bed, 6 ml reservoir.	Biotage	500-0010-C
Formic acid	Sigma	F0507-500ML
Ammonium acetate	Sigma	A1542-250G

(*Continued on next page*)

Continued

REAGENT or RESOURCE	SOURCE	IDENTIFIER
Critical Commercial Assays		
Alpha Trak 2 glucose meter	Zoetis	N/A
Insulin Elisa (Mouse Clamp studies)	Crista Chem	90080
NEFA assay (Mouse clamp studies)	WAKO	R1 436-91995
		R2 434-91795
		S: 270-77000
Triglyceride assays	Siemens Healthcare Diagnostics	N/A
Roche Free Fatty Acid Kit (half-micro test)	Roche	1138175001
Insulin ECL	Meso Scale Discovery	K152BZC
Deposited Data		
Raw data	This paper	https://data.mendeley.com/datasets/whn4s7nyww/draft?a=22693287-b601-457c-85a9-9903c6d88639; Reserved: https://doi.org/10.17632/whn4s7nyww.2
Experimental Models: Organisms/Strains		
Mouse: C57BL/6J	The Jackson Laboratory	JAX: 000664
Mouse: PPARy2 KO C57BL/6J.sv129	(Medina-Gomez et al., 2005)	N/A
Oligonucleotides		
PPARg2 F CCAACCAATCTTTTGCAAGACATAGAC	(Medina-Gomez et al., 2005)	N/A
PPARg2 R ACATGCAATTTCACCCACACATGAGTG	(Medina-Gomez et al., 2005)	N/A
PPARg2 Asc AATGGCCGCTTTTCTGGATTCATCGAC	(Medina-Gomez et al., 2005)	N/A
Sybr Green Primer and TaqMan primer and probe sequences see Table S1	N/A	N/A
GLUT4 TaqMan primer and probe set (Human)	ThermoFisher	Hs00168966_m1
Human PPIA (Cyclophilin A) Endogenous Control (FAM/MGB probe, non-primer limited)	ThermoFisher	4333763F
IRS1 TaqMan primer and probe set (Human)	ThermoFisher	Hs00178563_m1
Software and Algorithms		
IBM SPSS Statistics 22	IBM corporation	N/A
MassHunter Workstation Software Quantitative Analysis (Version B.07.00)	Agilent Technologies Inc	N/A

CONTACT FOR REAGENT AND RESOURCE SHARING

Further information and requests for resources and reagents should be directed and will be fulfilled by Dr. Samuel Virtue (sv234@medschl.cam.ac.uk).

EXPERIMENTAL MODEL AND SUBJECT DETAILS

Animals

PPARγ2 KO mice were generated as described previously (Medina-Gomez et al., 2005). Mice were phenotyped on a mixed C57BL/6/sv129 genetic background. Mice homozygous for a deletion in PPARγ2 (PPARγ2 $-/-$) and their wild-type littermates were generated by mating heterozygous mice. All animals used were male mice. Ages are provided in figure legends. C57BL/6 wild-type mice were purchased from Charles River UK. This research has been regulated under the Animals (Scientific Procedures) Act 1986 Amendment Regulations 2012 following ethical review by the University of Cambridge Animal Welfare and Ethical Review Body (AWERB). Animals were housed in a specific pathogen free facility with 12 hour light and 12 hour dark cycles. Unless otherwise stated all animals were studied under fed conditions and at 24°C.

Rodent Diets

Mice were fed either standard breeders chow (Safe Diets, DS-105) or a 45% fat diet (HFD) (Research Diets D12451) all diets were provided ab libitum.

Feeding regimes

For the one day high-fat diet challenge mice were single housed 1 week prior to the start of the diet to permit food intake assessment. The diet commenced in a staggered manner between 10 am and midday. Mice were alternated in genotype for both feeding of the diet and also for culling. Mice were killed in the same sequence that they were fed the diet in to keep the mice as close to receiving high-fat diet for 24 hours as possible. No significant correlation between the order the mice were killed in and the levels of FFAs, insulin or TG in blood was detected for the time course of the experiment. 1 month and 1 week HFD fed animals mice were culled between 10 am and 12pm in order to maintain the animals in the same stage of the circadian cycle as the chow controls and the 1 day high-fat fed animals

Patient recruitment

Forty five subcutaneous adipose tissue (SAT) samples from morbidly obese (BMI > 35 kg/m^2) participants with different degrees of insulin action (measured using hyperinsulinaemic–euglycaemic clamp) were analyzed. All these participants were of Caucasian origin, reported that their body weight had been stable for at least three months before the study and were studied in the post-absorptive state. BMI was calculated as weight (in kg) divided by height (in m) squared. Patients had no systemic disease other than obesity and all were free of any infections in the previous month before the study. Liver diseases (specifically tumoral disease and hepatitis C virus infection) and thyroid dysfunction were specifically excluded by biochemical work-up. All these participants were recruited at the Endocrinology Service of the Hospital of Girona 'Dr Josep Trueta'. All participants gave written informed consent, validated and approved by the Ethics Committee of the Hospital of Girona 'Dr Josep Trueta', after the purpose of the study was explained to them.

METHOD DETAILS

Glucose tolerance test

For fasted glucose tolerance tests mice were fasted overnight from 4 pm until 9 am the next day and then allowed to acclimatise to the procedural room for 1 hour. For fed glucose tolerance tests mice were allowed to acclimatise to the procedural room for one hour between 9 and 10 am. Glucose was administered at 1 g/kg IP. A fixed dose of glucose was given to all mice in the study group based on the average weight of the group. Fed glucose tolerance tests were performed at 10 am in the morning. Blood samples were collected at 0, 10, 20, 30, 60, 90 and 120 minutes.

For stable isotope GTTs mice were injected with a 50:50 mix of D2-6,6 Glucose and D2-2 Glucose (Goss Scientific, UK). Blood was collected on blood spot cards. A 6 mm punch was removed from each blood spot card and extracted by wetting with 40 ul of water followed by addition of 400 ul of ethanol. Samples were shaken for 45 minutes at room temperature before undergoing centrifugation at 16,000 g. EtOH extracts were dried under vacuum at ambient temperature using a Speed Vac. Extracts underwent aldonitrile pentaacetate derivitisation as follows: Dried extracts were resuspended in 50 ul of pyridine containing 2% w/v hydroxylamine. Samples were heated for 45 minutes at 90°C then allowed to cool. After cooling 100 ul of acetic anhydride was added and samples were heated for 30 minutes at 60°C for 30 minutes. Samples were allowed to cool and then dried under nitrogen at 60°C. Dried samples were dissolved in 200 ul of ethyl acetate, centrifuged at 16,000 g for 1 minute and the top 150 ul of supernatant was transferred to an autosampler vial containing a 300 ul glass insert. Gas-Chromatography Mass spectrometry analysis was performed using an Aglient 7890B GC and an Agilent 5977A MSD using a 0.25 uM x 30 M DB-5 (Cat: 122-5031, Agilent) column. The GC-Conditions were as follows:

Inlet conditions

Inlet temperature: 250°C
Split 20:1
Inlet liner Liner, ultra inert, splitless, single taper, glasswool (cat#6322616) (Agilent Technologies)
Column Flow 1 ml/min

Temperature Program:

80°C Hold for 1 minute
20°C/min until 280°C
280°C Hold for 3 minutes
MSD transfer line 290°C

MSD conditions:

Scan 45-850, 4 Hz
MS Source temperature 230°C
MS Quad temperature 150°C

Analysis was performed using MS Quantitative analysis. Ions analyzed were 217 (M0) 218 (M1), 219 (M2) 220 (M3) and 221 (M4). To determine the relative ratio of endogenous to exogenous glucose blood samples were compared to the glucose composition of mouse blood from animals uninjected with labeled glucose and a sample of D2-6,6 glucose (10 ul, 10mM) that was dried onto a blood spot card and extracted as described above). The ion pattern of each blood sample was compared to the tracer and endogenous blood glucose. To determine the relative contribution of endogenous and tracer glucose we defined 5 equations for each ion as follows:

$$\begin{aligned}
&\text{Equation System 1:}\\
&\text{M0calc} = x * \text{M0Endo} + (1\text{-}x) * \text{M0Exo}\\
&\text{M1calc} = x * \text{M1Endo} + (1\text{-}x) * \text{M1Exo}\\
&\text{M2calc} = x * \text{M2Endo} + (1\text{-}x) * \text{M2Exo}\\
&\text{M3calc} = x * \text{M3Endo} + (1\text{-}x) * \text{M3Exo}\\
&\text{M4calc} = x * \text{M4Endo} + (1\text{-}x) * \text{M4Exo}
\end{aligned}$$

Where x represents the Fractional concentration of the endogenous glucose in the blood and 1-x the Fractional concentration of exogenous glucose in the blood.

We defined a further 5 equations:

$$\begin{aligned}
&\text{Equation System 2:}\\
&\text{M0obs-M0calc}\\
&\text{M1obs-M1calc}\\
&\text{M2obs-M2calc}\\
&\text{M3obs-M3calc}\\
&\text{M4obs-M4calc}
\end{aligned}$$

We then used the Solver function of Excel to minimize the sum of the squares of the result of the 5 equations by changing the value of x in equation system 1 using the GRG Non-Linear Engine.

The solution for x defined the fractional concentration of endogenous glucose and 1-x the fractional concentration of D2-6,6 glucose. As D2-6,6 glucose represented 50% of the glucose injected in the GTT we multiplied the fractional concentration of D2-6,6 glucose by 2 to get the fractional concentration of the exogenous glucose and multiplied it by the observed blood concentration of glucose to get the concentration of exogenous glucose in mMol. By subtraction the exogenous glucose from the mouse's total blood glucose concentration we obtained the endogenous glucose concentration.

Mouse Euglycaemic-hyperinsulinaemic clamp

Animals were fasted from 4 pm until 8 am the next morning. Animals were anaesthetised with 11 μl per gram of body weight using a combination of the following drugs at stated concentrations fentanyl (0.03125 mg/ml), midazolam (0.625 mg/ml), and acepromazine (0.625 mg/ml). Once unconscious mice were placed on temperature controlled heating pads (Harvard Apparatus) and core body temperature was maintained at 37°C. Mice underwent a basal infusion period. The basal infusate comprised of 3-3H glucose (0.23 MBq/ml) and 3 mg/ml sodium citrate in 0.9% saline. The basal infusion rate was 100 ul/hour and was conducted for 90 minutes. For the clamp phase the basal infusate was stopped and the hyperinsulinaemic infusion was started. The hyperinsulinaemic infusate comprised of 0.11 mU per ml of insulin, 0.231 MBQ/ml of 3-3H glucose, 3 mg/ml sodium citrate, 2% FFA-free low-endotoxin BSA in 0.9% saline. The hyperinsulinaemic infusion was initiated with a bolus of 30 ul of infusate followed by 100 ul/hour. A variable rate infusion of glucose was then started using 12.5% glucose in 0.9% saline with 3 mg/ml sodium citrate. Blood glucose was measured every 5 minutes and glucose infusion rate was adjusted to clamp mice to their basal glucose levels. The hyperinsulinaemic infusion was continued for at least 90 minutes, or if mice had not reached steady state until steady state was achieved. Steady state was defined as mice exhibiting blood glucoses that were ± 0.5 mMol of their starting blood glucose and having a stable glucose infusion rate for three consecutive measurements. Blood samples were collected for the determination of FFA, Insulin and serum specific activity at the end of the basal and clamped states.

Mouse 2-Deoxyglucose uptake

Mice were fed a high-fat diet for 1 day, with mice placed on the diet between 8 and 9 am. The following day mice were anaesthetised in the sequence they were anaesthetised between 8 and 9 am. The anesthetic was 11 μl per gram of body weight using a combination of the following drugs at stated concentrations fentanyl (0.03125 mg/ml), midazolam (0.625 mg/ml), and acepromazine (0.625 mg/ml). Mice were injected IV with 0.046 MBq of 14C 2-Deoxyglucose and blood glucose levels were measured and serum samples collected at 10,20 and 30 minutes post injection in order to determine the specific activity of glucose in the blood. Mice were killed 30 minutes after injection with 14C-2-Deoxyglucose and tissues were harvested.

Phospho deoxyglucose was separated from deoxyglucose using solid-phase extraction with strong anion exchange columns. Tissues were weighed (~100 mg of WAT and ~50 mg of BAT) and homogenized in 1 mL of distilled water in glass tubes. The homogenates were heated to 95°C for 10 minutes before cooling to room temperature. Homogenates were then transferred to eppendorf tubes and centrifuged at 16,000 G and 66 ul of homogenate was taken for scintillation counting. SAX-SPE columns were conditioned

with 5ml of distilled water and then collection vials placed below the columns before transferring 660 ul of homogenate to the column. Columns were washed 3 times with 2 mL of distilled water. The wash fractions were vortexed and 500 ul of the wash fraction was added to 20 mL of scintillant for counting. New collection columns were placed under the SPE columns and the phosphodeoxyglucose was eluted three times using 2 mL of 0.2 M formic acid and 0.5 M ammonium acetate pH 5.0. The elution fractions were vortexed to mix them and 500 ul was added to 20 mL of scintillant and counted immediately.

Calorimetry and assessment of metabolic flexibility

Mice underwent calorimetry in a custom built calorimetry system (Creative Scientific UK) for up to 48 hours. Carbon dioxide and oxygen concentrations were determined every 11 minutes for each chamber and the incurrent air supply. Flow rates were 400 ml/minute. Energy expenditure was calculated from the VO2 and CO2 according to the modified Weir equation (EE J/min = 15.818xVO2 (ml/min) + 5.176*VCO2 (ml/min)). Metabolic flexibility was assessed by measuring the amplitude of respiratory exchange ratio (RER) from mice in free living calorimetry chambers. Amplitude of RER was defined as the difference between the average of the lowest 10% of RER values subtracted from the average of the highest 10% of RER values (dRER). Amplitude of RER has been established as a read out of metabolic flexibility by multiple groups (Asterholm et al., 2012; Asterholm and Scherer, 2010; Gao et al., 2015; Isken et al., 2010; Jackowski and Leonardi, 2014; Longo et al., 2008; Muoio et al., 2012; Riachi et al., 2004; Vaitheesvaran et al., 2010; Vroegrijk et al., 2013).

Lipid tolerance test

Mice were fasted overnight from 4 pm until 9 am the next day. Mice were gavaged with 200 μl olive oil (Heritage, NISA, UK). Blood samples were collected at 0,1,2,4 and 8 hours post gavage for determination of TG and FFA levels. See Serum biochemistry for details of TG and FFA measurement.

Liver fat %

100-200 mg of liver was weighed and lipids extracted according to the Folch extraction procedure (Folch et al., 1957). Briefly, 100 mg of liver was homogenized in 1 mL volumes (w/v) of 2:1 chloroform methanol using a MP Biomedical Fast Prep. Homogenized samples were centrifuged (16,000 G for 10 minutes) and the supernatant transferred to a clean Eppendorf tube. 200 ul of water was added and samples vortexed for 2 minutes. Samples were centrifuged (16,000 G for 10 minutes) and the lower organic phase was collected into a pre-weighed Eppendorf tube. A second round of extraction was carried out by adding 700 ul of chloroform to the sample and vortexing (2 minutes) and centrifuging (16,000 G for 10 minutes). The lower organic phase was collected again and combined with the previous extract. The extract was then dried under nitrogen. The weight of the tube and extracted lipid was measured and the weight of the empty tube subtracted to give the quantity of lipid in the sample. The liver fat extract was expressed as a % of wet liver weight.

Serum Biochemistry

Triglycerides were measured on the Dimension RXL analyzer (Siemens Healthcare). Free Fatty Acids were measured using the Roche Free Fatty Acid Kit (half-micro test) (kit code 11383175001). Insulin, was measured using electrochemical luminescence immunoassay on the MesoScale Discovery immunoassay platform.

RNA extraction and real time PCR

RNA was extracted using STAT-60 (AMS biotech) according to manufacturer's procedures. Reverse transcription (500 ng of total RNA) was performed using random hexamers using the Reverse Transcriptase System (Promega) according to manufacturer's instructions. Real-time PCR was carried out in 13ul reactions using TaqMan or Sybr Green reagents and performed in a 384 well plate on an ABI7900 real-time PCR machine using default thermal cycler conditions. For PCR primer sequences see Table S1.

Western Blotting

Protein was extracted from tissues using a modified RIPA buffer (50 mM Tris HCL pH 8, 150 mM NaCl, 1% NP-40, 0.5% Sodium Deoxycholate, 0.1% SDS) and quantified by the BioRad DC protein assay. Protein was heated with loading dye containing DTT and heated to 95oC for 5 minutes to denature proteins before loading. 10 ug per well of protein was and subjected to SDS-PAGE in a 4%–12% gradient gel using the Novex NuPage midi system (Life Technologies) and transferred using the iBlot transfer system and reagents (Life Technologies) for 7 minutes. Membranes were probed for: pHSL S660 (Cat: 4126 Cell Signaling, USA) total HSL (Cat: 4107 Cell Signaling, USA), Phospho-p44/42 MAPK (Cat: 4376 Cell Signaling, USA), total p44/42 MAPK (Cat: 4695 Cell Signaling, USA), Phospho AKT (Cat: 4060 Cell Signaling, USA), total-AKT (Cat: 2920 Cell Signaling, USA) and B-actin (Cat: 8226 Abcam, UK).

Histological analysis of white adipose tissue

Adipose tissue was fixed in formalin. Tissues were embedded in paraffin and sections were cut at a thickness of 100 μM. Slides were imaged using an automated slide scanning microscope (Axioscan Z1 using a Hamamatsu orca flash 4.0 V3 camera) using a 20x objective with a numerical aperture of 0.8. Adipocytes size was quantified using Halo (Indica Labs inc) utilizing the Vacuole module v.1.4. The cut off point for minimum vacuole diameter was set at 15 and the maximum at 150 μm. Adipocyte, blood vessels and slide background were determined using the tissue classifier method, only adipocytes were quantified.

GC-MS FAME analysis

Lipid extraction and analysis were described in detail previously (Yew Tan et al., 2015). Briefly, lipids were extracted by the Folch extraction procedure. Lipids were esterified to form methyl esters using BF3 methanol. Methyl esters were analyzed by Gas-chromatography Mass-Spectrometry. The identity of species was determine by retention time and mass spectra and compared to a food industry fame standard (Thames Restek, UK). Lipid species were quantified based on integrated peak area.

Lipid uptake to adipose tissue

Mice were clamped as described above. 30 minutes from the end of the clamp a bolus of 0.2 MBQ of 1-14C palmitate conjugated to 2% BSA in PBS was injected IV. After 30 minutes serum and adipose tissue was collected. Serum DPMs were measured directly. Adipose tissue was weighed and lipids extracted using the Folch method (see "Liver fat%" above). Lipids were separated into Neutral Lipid, Phospholipid and FFA fractions by solid phase extraction. Solid phase extraction was performed by resuspending the adipose tissue extract in 1 mL of dry chloroform. Of this 1 mL 100 ul was reserved for scintillation counting and 900 ul was applied to the SPE column and the flow through collected. Two further washes of 1 mL of dry chloroform were performed and combined with the initial flow through; this represented the neutral lipid fraction. Phospholipids were then eluted from the columns into a fresh tube using 2 mL of Chloroform:Methanol 60:40). The NEFA fraction was eluted in 2.0ml chloroform/methanol/glacial acetic acid (100:2:2). All SPE steps were performed using a vacuum manifold. Unlike many SPE lipid extraction protocols, no activation of the columns was required and between collection of fractions the bed was dried under vacuum for 2-3 s to minimize carry over between fractions.

LC-MS analysis

Sample extraction

The pre-weighed muscle tissue samples (~10-20 mg) were transferred to a 2 mL Eppendorf screw cap tubes. A single 5 mm stainless steel ball bearing and 400 μL of the chloroform:methanol mix solution (2:1, respectively) were added to the tissue samples, followed by a 3 s cycle of vigorous vortexing. The tissues were then homogenized in the extraction solvent using a Bioprep-24-1004 homogenizer (Allsheng, Hangzhou City, China) run at speed; 6 m/s, time; 30 s for 2 cycles. Following the homogenization, 150 μL of the stable isotope labeled lipid internal standard mix was added along with 10 μL the stable isotope 5-hydroxytryptamine-d4 acylcarnitine internal marker. The mixtures was then thoroughly vortexed before the addition of 600 μL of the chloroform:methanol mix solution (2:1, respectively). The samples were homogenized again (speed; 6 m/s, time; 30 s for 2 cycles). To the homogenates, 400 μL of HPLC-water was added with an additional cycle of vortexing. The samples were then centrifuged (5 mins at ~21,000 g) to produce aqueous and organic biphasic extracts. The two phases were then separated by pipetting each into separate 2 mL Eppendorf screw cap vials making sure not to break up the undissolved protein pellet. To perform a double extraction, 1 mL of the chloroform:methanol mix solution (2:1, respectively) was added to each sample, then vortexed thoroughly. The samples were then homogenized on the tissuelyser (speed; 6 m/s, time; 30 s for 2 cycles). Then 400 μL of HPLC-water was added to each sample, vortexed and homogenized on the tissuelyser (speed; 6 m/s, time; 30 s for 2 cycles). The samples were then centrifuged (5 mins at ~21,000 g) to produce the biphasic extracts, followed by phase separation into the corresponding 2 mL Eppendorf screw cap vials containing the first extracts.

Acylcarnitine sample preparation

Half of the corresponding organic fractions and half the aqueous fraction were mixed with each other into separate 2 mL Eppendorf screw cap vials. The mixed extracts were dried completely under a gentle stream of oxygen free nitrogen heated to 45°C. To the dried extracts, 200 μL of water:acetonitrile (4:1, respectively) was added to reconstitute the acylcarnitine species. The samples were then vortexed thoroughly. The samples were then transferred into 2 mL amber glass vials containing a 300 μL vial-insert which were then analyzed by liquid chromatography with mass spectrometry detection.

Acylcarnitine LC-MS

Chromatographic separation was achieved using an ACE Excel 2 C18-PFP (150mm * 2.1mm, 2 μm) LC-column with a Shimadzu UPLC system (Shimadzu UK Limited, Wolverton, Milton Keynes). The column was maintained at 55°C with a flow rate of 0.5 mL/min. A binary mobile phase system was used with mobile phase A; water (with 0.1% formic acid), and mobile phase B; acetonitrile (with 0.1% formic acid). The gradient profile was as follows; at 0 minutes_0% mobile phase B, at 0.5 minutes_100% mobile phase B, at 5.5 minutes_100% mobile phase B, at 5.51 minutes_0% mobiles phase B, at 7 minutes_0% mobile phase B. Mass spectrometry detection was performed on a Thermo Exactive orbitrap mass spectrometer (Thermo Scientific, Hemel Hempstead, UK) operating in positive ion mode. A heated electrospray source was used; the sheath gas was set to 40 (arbitrary units), the aux gas set to 15 (arbitrary units) and the capillary temperature set to 250°C. The instrument was operated in full scan mode from m/z 75–1000 Da. Acylcarnitine species were identified by detecting a signal peak for the corresponding accurate mass at the correct retention time. Signals were normalized to the dried tissue mass (dried protein pellet left after the extraction), their final semiquantitative concentrations were determined by comparing the acylcarnitine species intensity to the internal marker intensity.

Lipid sample preparation, 50 μL of the organic fractions were transferred into 2 mL amber glass vials containing a 300 μL vial-insert. The samples were then dried completely under a gentle stream of oxygen free nitrogen at 45°C. Then, 90 μL of isopropanol:acetonitrile (4:1, respectively) was added to reconstitute the lipid species. The samples were then vortexed thoroughly before being analyzed by liquid chromatography with mass spectrometry detection.

Lipid LC-MS, Chromatographic separation was achieved using a Waters Acquity UPLC CSH C18 (50mm * 2.1mm, 1.7 μm) LC-column with a Shimadzu UPLC system (Shimadzu UK Limited, Wolverton, Milton Keynes). The column was maintained at 55°C with a

flow rate of 0.5 mL/min. A binary mobile phase system was used with mobile phase A; acetonitrile:water mix (6:4, respectively, with 10 mM ammonium formate), and mobile phase B; isopropanol:acetonitrile mix (9:1, respectively, with 10 mM ammonium formate). The gradient profile was as follows; at 0 minutes_40% mobile phase B, at 0.4 minutes_43% mobile phase B, at 0.45 minutes_50% mobile phase B, at 2.4 minutes_54% mobile phase B, at 2.45 minutes_70% mobile phase B, at 7 minutes_99% mobile phase B, at 8 minutes_99% mobile phase B, at 8.3 minutes_40% mobile phase B, at 10 minutes_40% mobile phase B. Mass spectrometry detection was performed on a Thermo Exactive orbitrap mass spectrometer (Thermo Scientific, Hemel Hempstead, UK) operating in positive ion and negative ion continuous switching mode. Heated electrospray source was used; the sheath gas was set to 40 (arbitrary units), the aux gas set to 15 (arbitrary units) and the capillary temperature set to 300°C. The instrument was operated in full scan mode from m/z 150–1200 Da. Lipid species were identified by detecting a signal peak for the corresponding accurate mass at the correct retention time. Signals were normalized to the dried tissue mass (dried protein pellet left after the extraction), their final semi-quantitative concentrations were determined by comparing the lipid species intensity to the appropriate stable isotope labeled lipid internal standard.

Human Adipose tissue handling

Adipose tissue samples were obtained from SAT depots during elective surgical procedures (cholecystectomy, abdominal hernia surgery and gastric bypass surgery). Samples of adipose tissue were immediately transported to the laboratory (5-10 min). The handling of tissue was carried out under strictly aseptic conditions. Adipose tissue samples were washed in PBS, cut with forceps and scalpel into small pieces (100 mg) and immediately flash-frozen in liquid nitrogen before being stored at −80°C.

Hyperinsulinemic-euglycemic clamp in humans

After an overnight fast, two catheters were inserted into an antecubital vein, one for each arm, used to administer constant infusions of glucose and insulin, and to obtain arterialized venous blood samples. A 2-h hyperinsulinaemic-euglycaemic clamp was initiated by a two-step primed infusion of insulin (80mU/m^2/min for 5 min, 60mU/m^2/min for 5 min) immediately followed by a continuous infusion of insulin at a rate of 40mU/m^2/min (regular insulin (Actrapid, Novo Nordisk, NJ)). Glucose infusion began at minute 4 at an initial perfusion rate of 0.011 mmol/kg/min being then adjusted to maintain plasma glucose concentration at 4.9 – 5.5 mmol/l. Blood samples were collected every 5 minutes for determination of plasma glucose and insulin. Insulin sensitivity was assessed as the mean glucose infusion rate during the last 40 min. In the stationary equilibrium, the amount of glucose administered (M) equals the glucose taken by the body tissues and is a measure of overall insulin sensitivity.

RNA expression for human SAT

RNA purification was performed using an RNeasy Lipid Tissue Mini Kit (QIAgen, Izasa, Barcelona, Spain) and the integrity was checked using an Agilent Bioanalyzer (Agilent Technologies, Palo Alto, CA, USA). Gene expression was assessed by real-time PCR using a LightCycler 480 Real-Time PCR System (Roche Diagnostics, Barcelona, Spain), using TaqMan and SYBRgreen technology suitable for relative genetic expression quantification. The RT-PCR reaction was performed in a final volume of 12 μL. The cycle program consisted of an initial denaturing of 10 min at 95°C then 40 cycles of 15 s denaturizing phase at 95°C and 1 min annealing and extension phase at 60°C. A threshold cycle (Ct value) was obtained for each amplification curve and a ΔCt value was first calculated by subtracting the Ct value for human cyclophilin A (*PPIA*) RNA from the Ct value for each sample. Fold changes compared with the endogenous control were then determined by calculating $2^{-\Delta Ct}$, so gene expression results are expressed as expression ratio relative to PPIA gene expression according to manufacturers' guidelines. The commercially available and pre-validated TaqMan® primer/probe sets used were as follows: Peptidylprolyl isomerase A (cyclophilin A) (4333763, *PPIA* as endogenous control), insulin receptor substrate 1 (*IRS1*, Hs00178563_m1), solute carrier family 2 member 4 (*SLC2A4* or *GLUT4*. Hs00168966_m1). Human *PPARγ1* [forward: 5′-GGCCGCAGATTTGAAAGAAG-3′ and reverse: 5′-GGAGAGATCCACGGAGCTGAT-3′] and *PPARγ2* [forward: 5′-AAACCCCTATTCCATGCTGTTATG-3′ and reverse: 5′- TGTCAACCATGGTCATTTCTTGTG-3′] were measured using SYBR Green technology.

QUANTIFICATION AND STATISTICAL ANALYSIS

All statistics were performed using SPSS 25. Data points were excluded if they exhibit a value of more than two standard deviations from the mean. For all metabolic tests animals were randomly ordered into metabolic chambers (calorimetry) or to order in which experiments were conducted (GTT, Euglycaemic-hyperinsulinaemic clamps). Statistical significant was set at a p value of < 0.05. Specific tests are detailed in the figure legends.

DATA AND SOFTWARE AVAILABILLITY

https://data.mendeley.com/datasets/whn4s7nyww/draft?a=22693287-b601-457c-85a9-9903c6d88639

Cell Reports, Volume 24

Supplemental Information

Peroxisome Proliferator-Activated Receptor γ2 Controls the Rate of Adipose Tissue Lipid Storage and Determines Metabolic Flexibility

Sam Virtue, Kasparas Petkevicius, José Maria Moreno-Navarrete, Benjamin Jenkins, Daniel Hart, Martin Dale, Albert Koulman, José Manuel Fernández-Real, and Antonio Vidal-Puig

Supplementary Results

Supplemental Figures

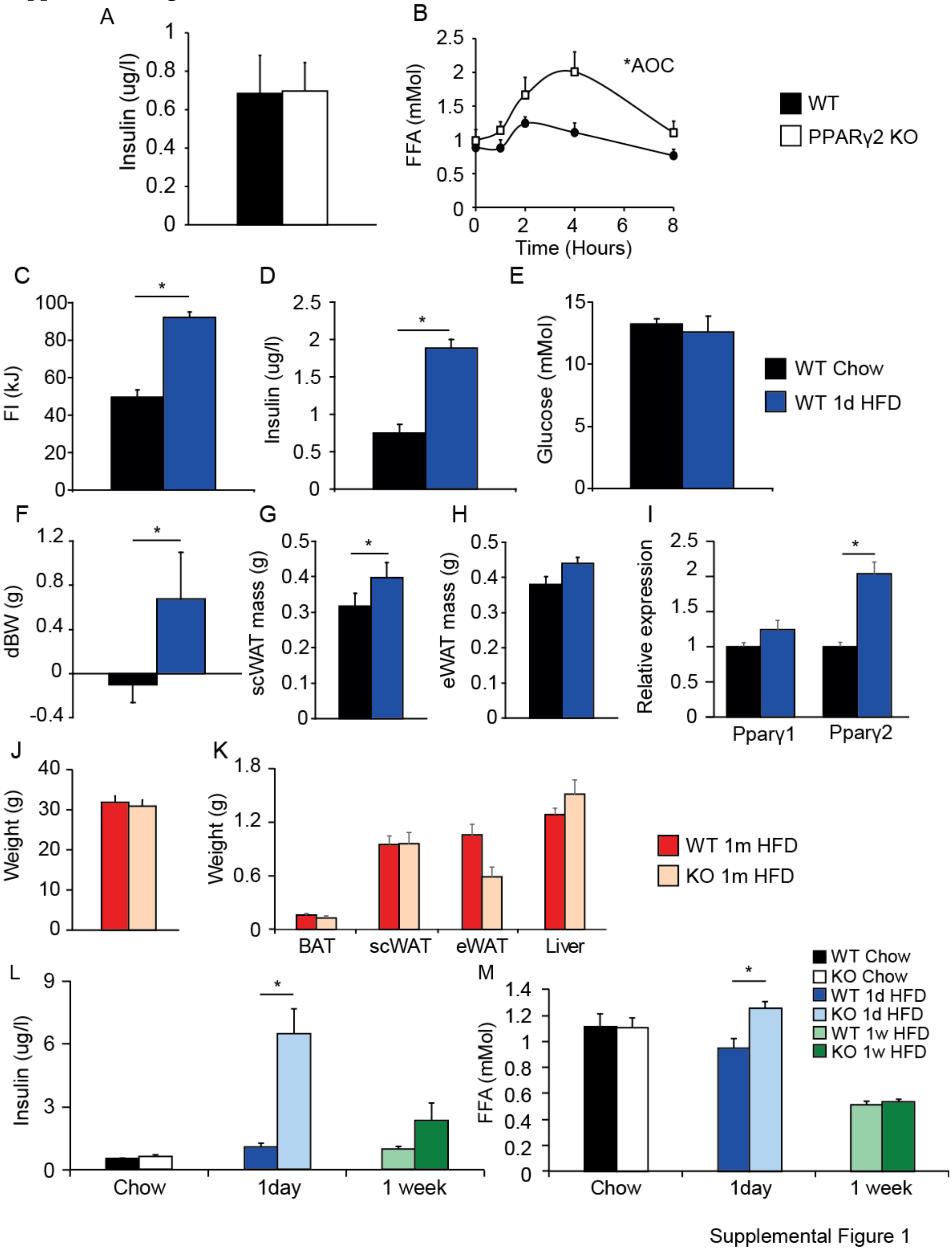

Figure S1 related to figures 1 and 2. A) Fasted insulin levels from chow fed PPARγ2 KO and WT mice N= 6 WT and 9 KO. B) FFA levels during a lipid tolerance test for WT and PPARγ2 KO mice. N=10 WT and 9 KO, 4 months old. *P<0.05 two-tailed students T-test. Metabolic and anthropomorphic characteristics of mice fed a high fat diet for one day (1dHFD) or chow controls; C) food intake (n=8 per group) D) serum insulin E) blood glucose (N=5 chow and 6 HFD) F) body weight change (dBW) G) subcutaneous white adipose tissue (WAT) mass H) epididymal WAT mass N=12 mice per group, C57bl/6 males, 10 weeks old. I) Gene expression in scWAT of 1dHFD fed mice or chow fed controls, N=6 mice per group, C57bl/6 males, 10 weeks old. J) Body weight and K) tissue weights for mice lacking PPARγ2 and wild-type controls following 1 month of high-fat feeding N=8 WT and 7 KO, 4 months of age. L) Insulin and M) FFA levels of a cohort of mice fed a chow control diet then switched to a high fat diet and blood samples measured after 0, 1 or 7 days of high-fat feeding. N= 7 mice per group, 3 months of age. All data represented as mean ±SEM.

Supplemental Figure 2

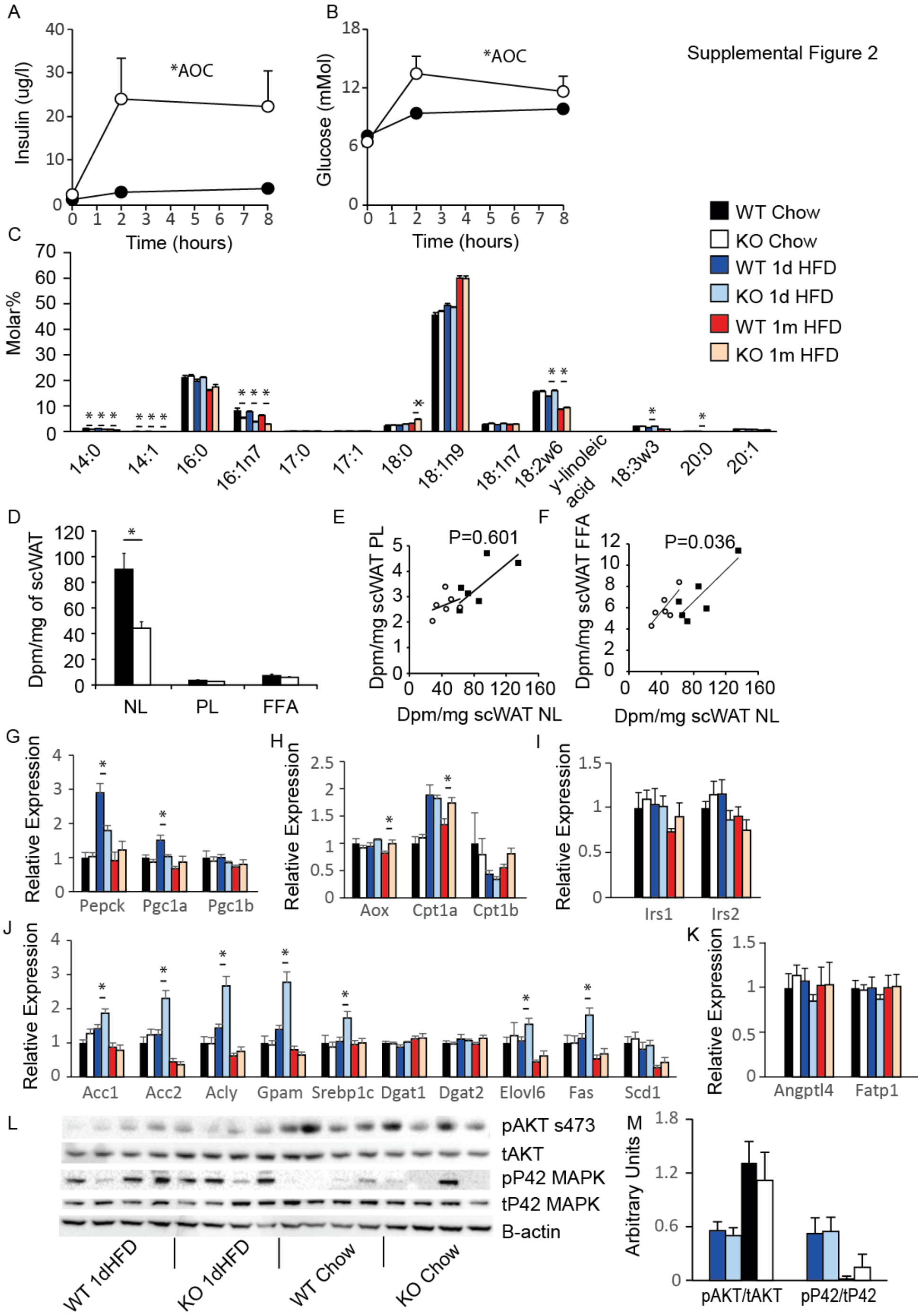

Figure S2 related to figures 2 and 3. A) Serum insulin and B) blood glucose for WT and PPARγ2 KO mice refed a T=0 following an overnight fast. N= 10 WT and 6 KO mice. C) Fatty acid methyl ester analysis by GC-MS for WT and PPARγ2 KO mice. N= 8 per group KO Chow, 1day HFD WT and KO, N=7 per group, WT Chow, 1month HFD WT and KO. D) Palmitate uptake and incorporation into different lipid fractions during a euglycaemic hyperinsulinaemic clamp in subcutaneous white adipose tissues expressed as absolute levels per mg of tissue. Relationships between palmitate incorporation into different fractions showing E) Phospholipid (PL) against neutral lipid (NL) F) Free fatty acids (FFA) against NL N =6 per group. Gene expression analysed by real-time PCR in the liver of chow, 1dHFD or 1 month high-fat fed wild type and PPARγ2 KO mice for: G) Gluconeogenic markers H) Fatty acid oxidative markers I) Insulin sensitivity markers J) lipid biosynthetic genes K) lipid uptake genes N=8 per group except 1 month HFD, N=6. Western blots of protein isolated from the Liver of PPARγ2 KO mice; L) representative western blots and M) quantification. N=8 mice per group for pAKT/tAKT, n=4 per group for pP42/tP42. For multiple time points 2-way ANOVA was performed, if significant pairwise comparisons were performed by two-tailed students T-test, *P<0.05. For determining differences in relationships between NL and PL and FFAs and ANCOVA model was used and reported p-values are for genotype effects. *P<0.05. All data represented as mean ±SEM.

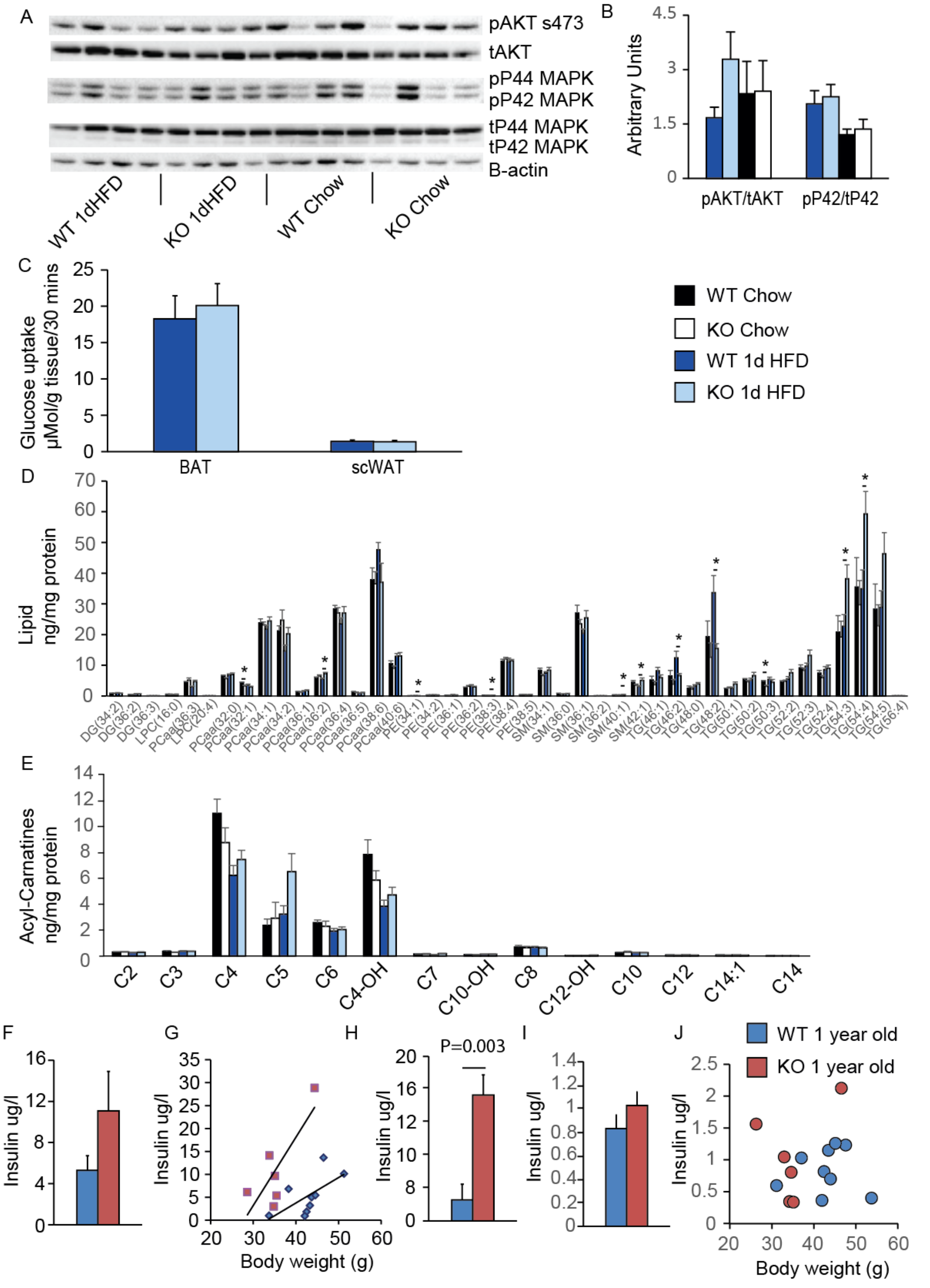

Supplemental Figure 3

Figure S3 related to figures 3 and 4. Western blots of protein isolated from the BAT of PPARγ2 KO mice; A) representative western blots and B) quantification. N=8 mice per group. C) Glucose uptake into BAT and scWAT of WT and PPARγ2 KO mice following 1dHFD. N=9 WT and 7 KO mice per group, 3 months old. Lipid profiles of skeletal muscle from WT and PPARγ2 KO mice fed either chow or 1dHFD showing D) neutral and phospholipids analysed by LC-MS E) acyl-carnatines analysed by tandem MS. N=6-8 per group *P<0.05, students T-test. All mice 4-5 months old. Serum insulin from 1 year old WT and PPARγ2 KO mice F) Raw values for fed insulin G) scatter plot of fed insulin against body weight showing a positive correlation H) Fed insulin levels corrected for body weight with ANCOVA. I) Raw values for fasted insulin J) Scatter plot of fasted insulin against body weight showing no correlation. N= 9 WT and N=6 KO, male mice, chow fed. Raw insulin values analysed by two-tailed students T-test. If body weight showed a significant correlation with insulin concentration in an ANCOVA model, estimated marginal means were reported. *P<0.05. All data represented as mean ±SEM.

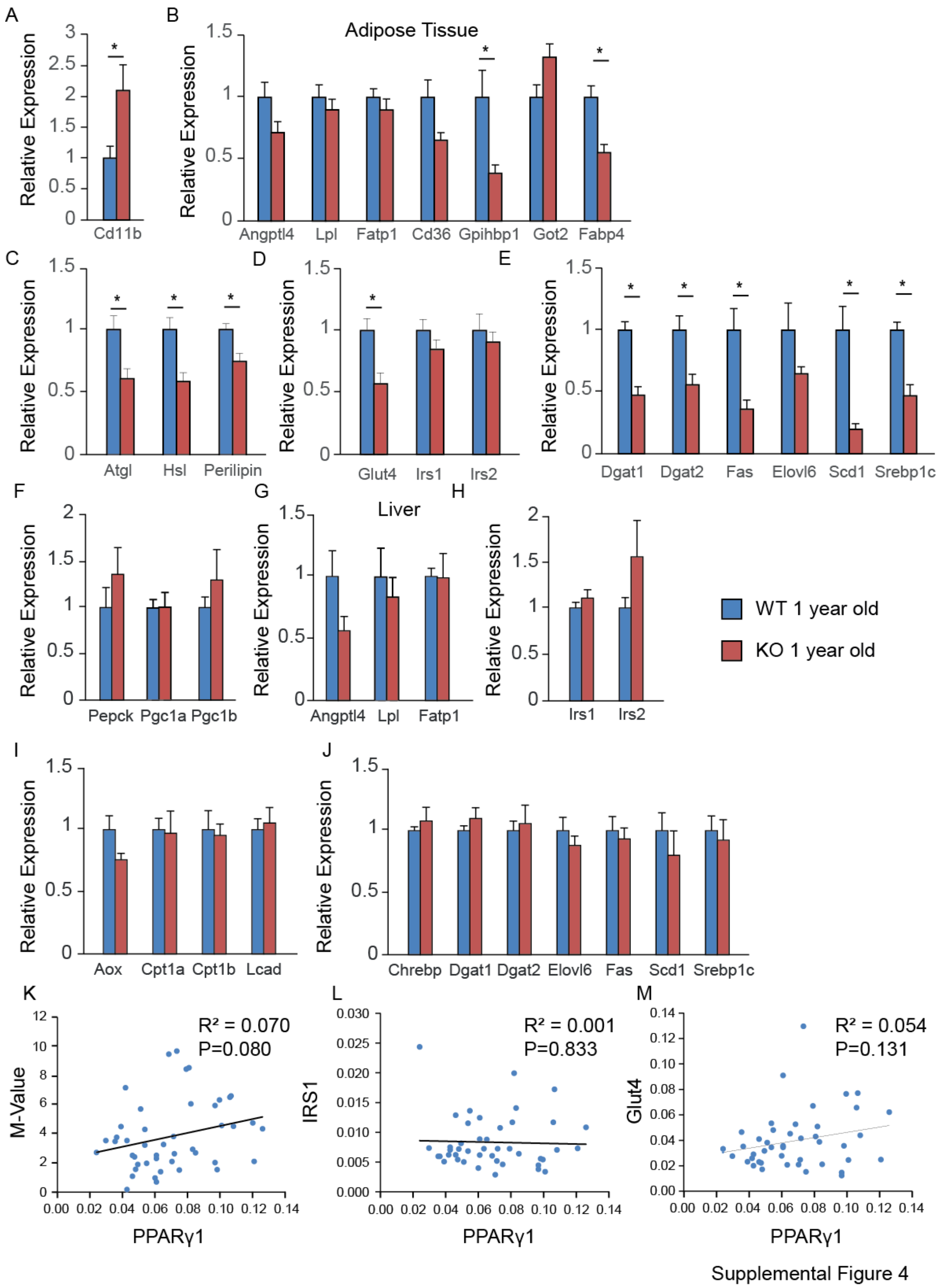

Figure S4 related to figure 4. Gene expression analysed by real-time PCR from PPARγ2 KO and WT mice aged to 1 year from adipose tissue A) Cd11b, B) Lipid uptake genes, C) lipolysis markers, D) insulin sensitivity markers and E) lipid biosynthetic genes. Gene expression from PPARγ2 KO and WT mice aged to 1 year from liver for F) Gluconeogenic markers G) Lipid uptake genes H) Insulin sensitivity markers I) Fatty acid oxidative genes J) Lipid biosynthetic and storage genes. N=10 WT, N=6 KO. *$P<0.05$ two-tailed students T-test. Correlations between PPARγ1 expression in human scWAT and K) M-Value during a clamp L) IRS1 expression and M) Glut 4 expression. N=45 subjects. Correlations are Pearson's, exact P-values are reported. All data represented as mean ±SEM.

Table S1. Real time PCR primer sequences relating to STAR methods

Gene code	Species	For	Rev	Probe
18S	Various	CGGCTACCACATCCAAGGAA	GTCGGAATTACCGCGGCT	GAGGGCAAGTCTGGTGCCAG
36B4	Mus musculus	AGATGCAGCAGATCCGCAT	GTTCTTGCCCATCAGCACC	
ACC1	Mus musculus	TGCAGATGACTTCCCTAACCTCTT	CCAGATGTCCGAGATTGTTTTGC	
ACC2	Mus musculus	CGAGGCCACGTCATTGC	CTGAAGTAACCCCACACGTTCTT	
ACLY	Mus musculus	TCACACTGCCAACTTCCTCCTTA	ACTCAGAAAAAGATGCTGTCCTACTG	
ALPHA ENOLASE	Mus musculus	CCCTCTTTCCTTGCTTTGCA	TGGCGTGGATCCTGAGAATAG	
ANGPTL4	Mus musculus	CAAGATGACCCAGCTCATTGG	ATCACTGTCCAGCCTCCATC	
AOX	Mus musculus	AATTGGCACCTACGCCCAG	AGTGGTTTCCAAGCCTCGAA	
AP2 (FABP4)	Mus musculus	CACCGCAGACGACAGGAAG	GCACCTGCACCAGGGC	TGAAGAGCATCATAACCCTAGATGGCGG
ATGL	Mus musculus	CGCCTCTCGAAGGCTCTCT	TGTAGCCCTGTTTGCACATCTC	
CAC	Mus musculus	GCATGTGCCTGGTGTTTGTG	CAGACAAACTTGGTGGTTGTGTCT	
CD11B	Mus musculus	CAGACAGGAAGTAGCAGCTCCT	CTGGTCATGTTGATGAAGGTGCT	
CD36	Mus musculus	GCCAAGCTATTGCGACATGA	TCTCAATGTCCGAGACTTTTCA	CACAGACGCAGCCTCCTTTCC
CHREBPA	Mus musculus	AGTGCTTGAGCCTGGCCTAC	TTGTTCAGGCGGATCTTGTC	
CPT1A	Mus musculus	CCTGGGCATGATTGCAAAG	GCCACTCACGATGTTCTTCGT	
CPT1B	Mus musculus	GCGTGCCAGCCACAATTC	TCCATGCGGTAATATGCTTCAT	CCGGTACTTGGATTCTGTGCGGCC
DGAT1	Mus musculus	AGGTTCTCTAAAAATAACCTTGCATT	TCGTGGTATCCTGAATTGGTG	
DGAT2	Mus musculus	GGCGCTACTTCCGAGACTAC	TGGTCAGCAGGTTGTGTGTC	
ELOVL6	Mus musculus	TGCAGGAAAACTGGAAGAAGTCT	ATGCCGACCACCAAAGATAAA	
F4/80	Mus musculus	CAGATACAGCAATGCCAAGCA	GATTGTGAAGGTAGCATTCACAAGTG	6TGCAGGGCAGGGATCTTGGTTATGC0
FAS	Mus musculus	GCCCAGACAGAGAAGAGGCA	CTGACTCGGGCAACTTCCC	6GGAGGAGGTGGTGATAGCCGGTATGTC0
FATP1	Mus musculus	CGTTTCGATGGTTATGTTAGTGACA	CATCACTAGCACGTCACCTGAGA	
GAPDHC	Mus musculus	AAGGTGGCAGAGGCCTTTG	TGCCCATTTAGCATCTCCTTCT	
GAPDHM	Mus musculus	AGCTGGTAGGCGCCATTG	GGGCGATGGCGAGGTT	
GLUT1	Mus musculus	GCTTCCTGCTCATCAATCGTAAC	CATCGGCTGTCCCTCGAA	
GLUT4	Mus musculus	ACTCATTCTTGGACGGTTCCTC	CACCCCGAAGATGAGTGGG	TGGCGCCTACTCAGGGCTAACATCA
GOT2	Mus musculus	TGGAGTCACAGCTGAAGATCTTG	CACCTCTTGCAACCATTGCTT	
GPAM	Mus musculus	CAATGGCGTACTTCATGTGTTCA	GCACCTCTTATTCAGGACTGCAT	
GPIHBP1	Mus musculus	CAGCAAACCCTTCTGCATCA	ACCACATGGAGTAGGTAGTCAGGTAA	
HSL	Mus musculus	GGAGCACTACAAACGCAACGA	TCGGCCACCGGTAAAGAG	CAGGCCTCAGTGTGACCGCCAGTT
IRS1	Mus musculus	TCCAGAAGCAGCCAGAGGA	AGGATTTGCTGAGGTCATTTAGGT	CAATAGCGTAACTGGACATCACAGCAGAATGAA
IRS2	Mus musculus	GCCTACACGCCTATCGCTAGA	CTCTTGGGCTCTGTGGGTAGA	CAGACCCTTCCCAGCATGCAAGAGTAC
LCAD	Mus musculus	GCATGAAACCAAACGTCTGGA	TGTTTTGTAATTCAGATGCCCAGT	TCCGGTTCTGCTTCCATGGCAAAA
LDH-H	Mus musculus	GCATGTGCCATCAGCATTCT	AGCTTGTCTTCCAACACATCCA	
LDH-M	Mus musculus	GCTGGGTCCTGGGAGAACAT	GACGCCGGCAACATTCAC	
LPL	Mus musculus	TGGAGAAGCCATCCGTGTG	TCATGCGAGCACTTCACCAG	TGCAGAGAGAGGACTCGGAGACGTGG
MCD	Mus musculus	CGGGACCTTCCTCATAAAGAGA	GATAGGCGACAGGCTTGAAAA	
PEPCK	Mus musculus	TGTGGGCGATGACATTGC	TGGCATTTGGATTTGTCTTCAC	6TATCAACCCAGAAAACGGGTTTTTTG0
PERILIPIN	Mus musculus	TGGACCACCTGGAGGAAAAG	TTCGAAGGCGGGTAGAGATG	
PFK-L	Mus musculus	GCCACCATCAGCAACAATGT	GACTGCTTGATGCGATCACAA	
PFK-M	Mus musculus	GGAGATCGTAGACGCCATCAC	CGGCCCATCACTTCTAACACA	
PFK-P	Mus musculus	CCTTCGTCCTGGAGGTGATG	CGGCACCGCAAGTCAAG	
PGC1A	Mus musculus	AACCACACCCACAGGATCAGA	CTCTTCGCTTTATTGCTCCATGA	6CAAACCCTGCCATTGTTAAGACCGAGAA0
PGC1B	Mus musculus	GGCCTTGTGTCAAGGTGGAT	GGTGCTTATGCAGTTCCGTACA	AGACCCCCACACTGCGGGCTC
PPARG1	Mus musculus	TTTAAAAACAAGACTACCCTTTACTGAAATT	AGAGGTCCACAGAGCTGATTCC	6AGAGATGCCATTCTGGCCCCACCAACTT0
PPARG2	Mus musculus	GATGCACTGCCTATGAGCACTT	AGAGGTCCACAGAGCTGATTCC	6AGAGATGCCATTCTGGCCCCACCAACTT0
SCD1	Mus musculus	CTTGCGGATCTTCCTTATCATT	GATCTCGGGCCCATTCG	6ACCATGGCGTTCCAGAATGACGTGT0
SREBP1C	Mus musculus	GCCATGGATTGCACATTTGA	GGCCCGGGAAGTCACTG	GACATGCTCCAGCTCATCAACAACCAAG
PPARY1	Human	GGCCGCAGATTTGAAAGAAG	GGAGAGATCCACGGAGCTGAT	
PPARY2	Human	AAACCCCTATTCCATGCTGTTATG	TGTCAACCATGGTCATTTCTTGTG	
